# Seasonal Water Mass Evolution and Non‐Redfield Dynamics Enhance CO_2_ Uptake in the Chukchi Sea

**DOI:** 10.1029/2021JC018326

**Published:** 2022-08-04

**Authors:** Zhangxian Ouyang, Andrew Collins, Yun Li, Di Qi, Kevin R. Arrigo, Yanpei Zhuang, Shigeto Nishino, Matthew P. Humphreys, Naohiro Kosugi, Akihiko Murata, David L. Kirchman, Liqi Chen, Jianfang Chen, Wei‐Jun Cai

**Affiliations:** ^1^ School of Marine Science and Policy University of Delaware Newark DE USA; ^2^ NOAA Pacific Marine Environmental Laboratory Seattle WA USA; ^3^ Polar and Marine Research Institute Jimei University Xiamen China; ^4^ Key Laboratory of Global Change and Marine‐Atmospheric Chemistry of Ministry of Natural Resources Third Institute of Oceanography MNR Xiamen China; ^5^ Department of Earth System Science Stanford University Stanford CA USA; ^6^ Key Laboratory of Marine Ecosystem Dynamics Second Institute of Oceanography Ministry of Natural Resources Hangzhou China; ^7^ Institute of Arctic Climate and Environment Research Japan Agency for Marine‐Earth Science and Technology (JAMSTEC) Yokosuka Japan; ^8^ Department of Ocean Systems (OCS) NIOZ Royal Netherlands Institute for Sea Research Texel The Netherlands; ^9^ Meteorological Research Institute Tsukuba Japan; ^10^ Global Ocean Observation Research Center Research Institute for Global Change Japan Agency for Marine‐Earth Science and Technology (JAMSTEC) Yokosuka Japan

## Abstract

The Chukchi Sea is an increasing CO_2_ sink driven by rapid climate changes. Understanding the seasonal variation of air‐sea CO_2_ exchange and the underlying mechanisms of biogeochemical dynamics is important for predicting impacts of climate change on and feedbacks by the ocean. Here, we present a unique data set of underway sea surface partial pressure of CO_2_ (*p*CO_2_) and discrete samples of biogeochemical properties collected in five consecutive cruises in 2014 and examine the seasonal variations in air‐sea CO_2_ flux and net community production (NCP). We found that thermal and non‐thermal effects have different impacts on sea surface *p*CO_2_ and thus the air‐sea CO_2_ flux in different water masses. The Bering summer water combined with meltwater has a significantly greater atmospheric CO_2_ uptake potential than that of the Alaskan Coastal Water in the southern Chukchi Sea in summer, due to stronger biological CO_2_ removal and a weaker thermal effect. By analyzing the seasonal drawdown of dissolved inorganic carbon (DIC) and nutrients, we found that DIC‐based NCP was higher than nitrate‐based NCP by 66%–84% and attributable to partially decoupled C and N uptake because of a variable phytoplankton stoichiometry. A box model with a non‐Redfield C:N uptake ratio can adequately reproduce observed *p*CO_2_ and DIC, which reveals that, during the intensive growing season (late spring to early summer), 30%–46% CO_2_ uptake in the Chukchi Sea was supported by a flexible stoichiometry of phytoplankton. These findings have important ramification for forecasting the responses of CO_2_ uptake of the Chukchi ecosystem to climate change.

## Key Points

- Atmospheric CO_2_ uptake potential is larger in the nutrient‐rich non‐Alaska Coastal Water than the nutrient‐poor Alaska Coastal Water
- For the most intensive growing period (spring to early summer), net community production estimation was 66%–84% higher based on dissolved inorganic carbon (DIC) than ${\text{NO}}_{3}^{-}$
- A non‐Redfield C:N uptake ratio by phytoplankton enables more efficient DIC‐fixation and contributes 30%–46% of CO_2_ uptake

## 1 Introduction

As the first region of the Arctic Ocean to receive water and nutrient inputs from the Pacific Ocean, the Chukchi Sea is a unique ecosystem affecting Arctic biogeochemical cycles, food‐web function, and air‐sea CO_2_ fluxes (Bates et al., 2011; Grebmeier et al., 2015; Tremblay et al., 2015). The high biological productivity and the associated strong CO_2_ sink of the Chukchi Sea are primarily sustained by northward flowing, nutrient‐rich Pacific Water (Ardyna & Arrigo, 2020; Lewis et al., 2020; Ouyang et al., 2020; Tu et al., 2021). Woodgate (2018) reported that the annual mean flow of Pacific Water has increased by ∼50% between 1990 and 2015, bringing much more nutrients onto the Chukchi shelf, which has led to higher primary production and CO_2_ uptake in recent decades (Arrigo and van Dijken, 2015; Lewis et al., 2020). However, the increase of the annual mean transport of different water masses is not proportional (Woodgate, 2018), which implies that the Chukchi ecosystem may respond differently to the volume increase depending on the different properties of the incoming waters. For example, Lowry et al. (2015) suggested that the summer phytoplankton bloom was sustained by the nutrient‐rich winter water and that the persistent biological “hotspot” in the northern Chukchi Sea was driven by the flow and confluence of Pacific winter water rather than the summer Alaska Coastal Water (ACW). This was corroborated by a recent model study (Zheng et al., 2021). Counterintuitively, Strong et al. (2016) found that carbon export was larger in the less‐productive ACW than in other more productive water masses. Thus, possible changes of water masses and related changes in water column structure may, in return, affect biogeochemical processes in the Chukchi Sea.

In addition, the seasonal evolution of water masses associated with variations in biological processes plays a key role in controlling nutrients and carbon cycles in the Chukchi Sea. High primary production associated with Pacific Water inflow and sea ice retreats transforms the Chukchi Sea from a nutrient‐rich system in spring to a nutrient‐limited system in summer (Mills et al., 2015; Zhuang et al., 2020). Such a substantial change in nutrient availability potentially changes the uptake ratio of nutrients and carbon by phytoplankton to form particulate organic matter (POM) and dissolved organic matter (DOM) during primary production and complicates our understanding of the seasonal net community production (NCP) and CO_2_ uptake from the atmosphere in the Chukchi Sea (Tu et al., 2021; Zheng et al., 2021). Deviations of C:N uptake stoichiometry from the Redfield ratio have been widely observed over the world's oceans (Koeve, 2006; Martiny et al., 2013; Park et al., 2008). Several model studies have suggested that non‐Redfield stoichiometry of phytoplankton should be taken into account to better project the net primary production, food quality and ocean carbon uptake in response to climate change (Buchanan et al., 2018; Kwiatkowski et al., 2018). However, a variable C:N uptake stoichiometry within a seasonal scale is rarely investigated and it is much less known how a non‐Redfield C:N assimilation ratio of phytoplankton may affect NCP and CO_2_ uptake in a climate‐sensitive region such as the Chukchi Sea.

As the Chukchi Sea is one of the fastest changing regions in the world due to anthropogenic climate changes (Lewis et al., 2020; Meredith & Sommerkorn, 2019; Onarheim et al., 2018; Stroeve & Notz, 2018; Woodgate & Peralta‐Ferriz, 2021), it is important to better understand the fundamental drivers, causes, and patterns in seasonal biogeochemical dynamics. Although many efforts have been made to demonstrate the seasonal variation in CO_2_ fluxes and biogeochemistry in the Chukchi Sea (Ardyna & Arrigo, 2020; Lowry et al., 2015; Ouyang et al., 2021; Zheng et al., 2021), most of them were based on observations that were either within a single season or over multiple seasons across different years. Given that sea ice conditions in the Chukchi Sea vary from year to year, the timing and magnitude of physical and biological processes change correspondingly, perhaps causing a large uncertainty in deconvoluting the complicated relationship between seasonal air‐sea CO_2_ flux, NCP, and carbon and nutrient consumption using data collected across different years. Therefore, measurements of biogeochemical properties across multiple periods within a single year provide us a great opportunity to elucidate the links between seasonal evolution of water masses and the associated biogeochemical processes and ecosystem responses, which are essential for forecasting the responses of the Chukchi ecosystem to climate change.

Here, we present a data set of underway sea surface temperature (SST), salinity (SSS) and sea surface CO_2_ partial pressure (*p*CO_2_) measured in five consecutive cruises in the Chukchi Sea from spring to fall in 2014 (Table 1). We take advantage of this unique data set to examine both the spatial and seasonal variations in sea surface *p*CO_2_, and then explore the dominant drivers of seasonal *p*CO_2_ change along water mass evolution. We also use discrete sample‐based biogeochemical properties including DIC, total alkalinity (TA), nutrients (${\text{NO}}_{3}^{-}$ and ${\text{PO}}_{4}^{3-}$), dissolved oxygen (DO), and chlorophyll *a* (Chl *a*) from three cruises to identify how seasonal evolution of water masses affect biogeochemical dynamics. In addition, we calculated NCP based on seasonal changes in DIC and nutrients and discussed possible mechanisms for the observed inconsistency in the derived NCP values. Finally, we used a box model to evaluate the impacts of non‐Redfield C:N uptake by phytoplankton on seasonal CO_2_ uptake in the Chukchi Sea.

**Table 1:** Summary of Cruises Information and Data Sources

Cruise	Period	Vessel	Data source of sea surface *p*CO_2_	Data source of discrete samples
33HQ20140505	Start: 5 May 2014	Healy, USA	SOCATv2020	https://arcticdata.io/catalog/view/doi%3A10.18739%2FA21C1TG6R
	End: 21 June 2014			
33HQ20140709	Start: 9 July 2014 End: 2 August 2014	Healy, USA	SOCATv2020	No data collected
33HQ20140810	Start: 10 August 2014 End: 29 August 2014	Healy, USA	SOCATv2020	No data collected
76XL20140727	Start: 27 July 2014	Xuelong, China	SOCATv2020	https://data.mendeley.com/datasets/dfpxxwm24c/2
	End: 9 September 2014			
49NZ20140831	Start: 3 September 2014	Mirai, Japan	JAMSTEC	http://www.godac.jamstec.go.jp/darwin/cruise/mirai/mr14-05/e
	End: 28 September 2014			

## 2 Method

### 2.1 Study Area and Water Masses

**Figure 1:**
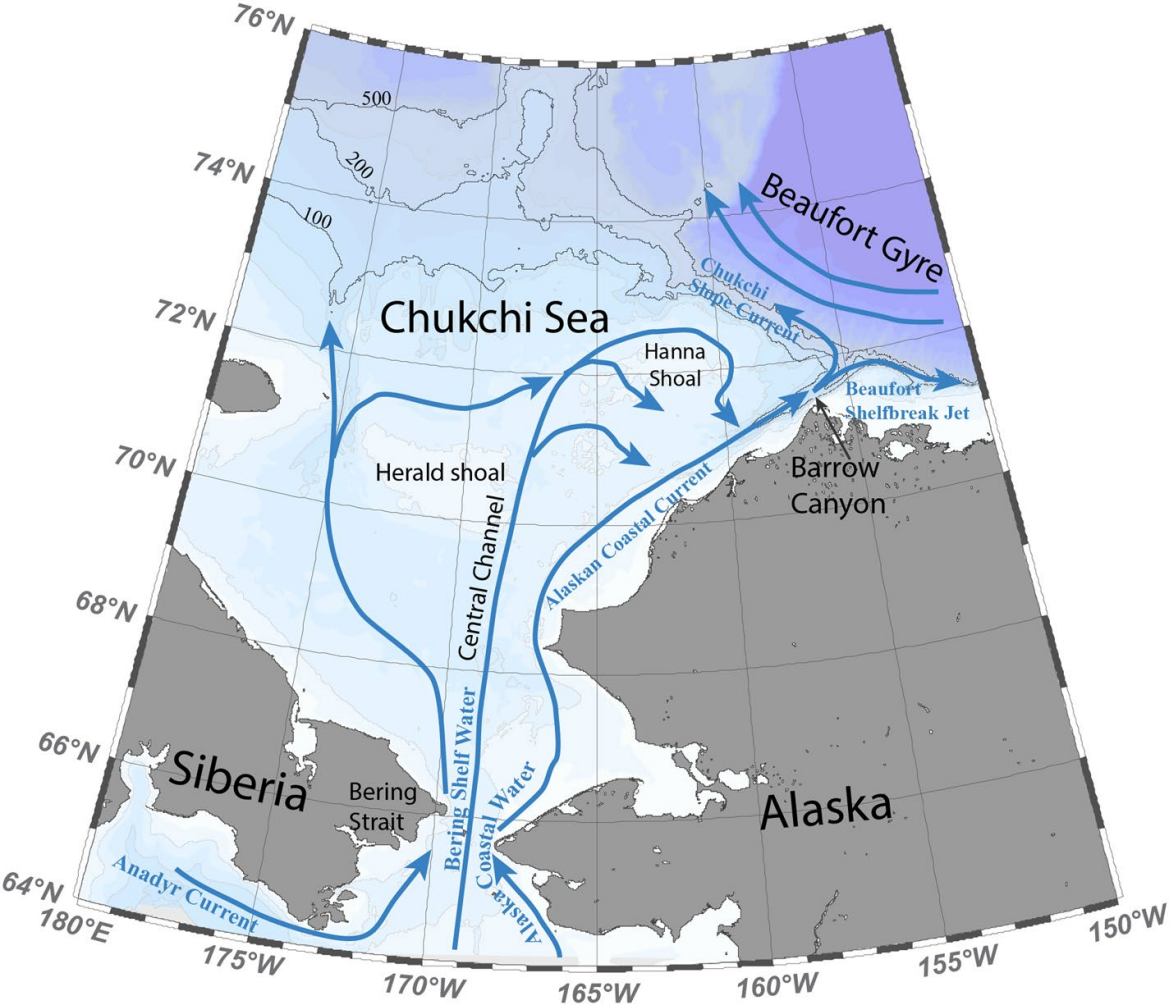
Schematic circulation of the Chukchi Sea and the names of associated geographical locations (after Corlett & Pickart, 2017).

The water mass properties and circulation in the Chukchi Sea have been extensively studied and presented in many previous works (Corlett & Pickart, 2017; Gong & Pickart, 2015; Li et al., 2019; Lin et al., 2019; Pacini et al., 2019; Pickart et al., 2019; Stabeno et al., 2018). Briefly, three main current branches of Pacific Water flow northward through Bering Strait onto the Chukchi Sea shelf (Figure 1). On the eastern side, the ACW branch flows along the Alaskan coast toward Barrow Canyon (known as the Alaskan Coastal Current (ACC) in summer and fall). The central branch flows through Central Channel between Herald Shoal and Hanna Shoal (Weingartner et al., 2005; Woodgate & Aagaard, 2005). The western branch transports Anadyr Water along the Siberian coast toward Herald Canyon (Weingartner et al., 2005). However, there is increasing evidence that the flows are not as rigidly confined to these three separate pathways as was originally thought. For example, a portion of western branch flows eastward to the north of Herald Shoal, where it meets the central branch before joining the eastern branch and exiting the shelf via Barrow Canyon (Figure 1). It is evident that topographic features such as canyons and shoals greatly affect the shelf circulation.

To focus on the Chukchi shelf, data used for this study were limited from 66°N to 74°N to a maximum of 120 m to exclude deep waters from the Canada Basin. We adopted the water mass classification used by several previous studies (Table 2; Corlett and Pickart et al., 2017; Gong & Pickart, 2015; Pacini et al., 2019; Pickart et al., 2019; Wang et al., 2022). The coldest water is known as Newly‐Ventilated Winter Water (NVWW; T < −1.6°C), which is typically observed from spring to early‐summer over the entire shelf. As summer progresses, this winter water can be converted to Remnant Winter Water (RWW; −1.6°C < T < 0°C) by solar heating or mixing with warmer summer water, which sometimes lasts into summer (Gong & Pickart, 2015). During summer and early fall, the Chukchi Sea mainly contains two Pacific summer waters. The first is ACW, which originates in the Gulf of Alaska. In the summer, the ACC carries predominantly ACW in the eastern Chukchi Sea, in which the water temperature can reach >10°C. The second is a combination of Anadyr Water and central Bering Shelf water, which is referred to as Bering Summer Water (BSW). The seasonal sea ice Melt Water (MW) forms a layer of fresh water on the Chukchi shelf. Sometimes it can also be divided into early‐season melt water and late‐season melt water (Table 2; Gong & Pickart, 2015; Pickart et al., 2019). This freshwater layer evidently occupies a large portion of the Chukchi/Beaufort shelfbreak regions in the mid‐to late‐summer. In these regions, the densest and saltiest water, the Atlantic Water (AW; T > −1.0°C and *S* > 33.6), can be observed at depths below ∼150 m, but it is rarely observed on the Chukchi shelf. However, AW can be upwelled occasionally through Barrow Canyon (Pisareva et al., 2019) and Herald Canyon (Pickart et al., 2010).

**Table 2:** Definition of Water Masses in the Chukchi Sea

Water masses		Temperature (°C)	Salinity
Newly‐ventilated Winter Water	NVWW	*T* < −1.6°C	*S* > 31.5
Remnant Winter Water	RWW	−1.6°C < T < 0°C	*S* > 31.5
Alaskan Coastal Water	ACW	*T* > 3.0°C	30 < *S* < 32
Bering Summer Water	BSW	0°C < T < 3.0°C	30 < *S* < 33.6
		*T* > 3.0°C	32 < *S* < 33.6
Early‐season Melt Water	ESMW	*T* < 0°C	*S* < 31.5
Late‐season Melt Water	LSMW	*T* > 0°C	*S* < 30
River Water	RW	*T* > 8.0°C	*S* < 30

### 2.2 Underway Measurements

It is unusual that there are five survey cruises in 1 year (2014) with underway sea surface temperature (SST), salinity (SSS) and *p*CO_2_ measured in the Chukchi Sea (Table 1). To our knowledge, this is the only year so far with such high‐density observations. The underway data were obtained from the SOCAT database (v2020; Bakker et al., 2016) and Japan Agency for Marine‐Earth Science and Technology (JAMSTEC) (Table 1). Specifically, the underway SST and SSS were measured using an underway water monitoring system in an intake port near the bow of the ships (5–7 m below the waterline). The underway sea surface *p*CO_2_ from USCGC Healy and RV Xuelong was measured using an underway *p*CO_2_ system (General Oceanic, USA) described in Pierrot et al. (2009). The data from RV Mirai was analyzed by a Greenhouse Gas Analyzer (Los Gatos Research, USA). These systems were monitored and calibrated with four certified gas standards (NOAA) every 2–3 hr. The overall precision of the reported sea surface *p*CO_2_ values was estimated to be ±2 μ atm. The underway *p*CO_2_ system and data reduction procedure were described by Pierrot et al. (2009).

### 2.3 Discrete Sample Analysis

Discrete biogeochemical samples were collected during three cruises (Table 1). The datasets of discrete samples included nutrients (NO_3_^−^ and PO_4_^3−^), dissolved oxygen (DO), Chl *a*, DIC and TA. The data, collected from USCGC Healy in spring, from RV Xuelong in summer, and from RV Mirai in fall, have been archived in publicly accessible data centers (Table 1).

In the spring cruise, nutrients, DO and Chl *a* were measured onboard. Nutrient samples were determined using a Seal Analytical continuous‐flow AutoAnalyzer 3 following a modified procedure after Armstrong et al. (1967). DO was measured using a spectrophotometer following the standard Winkler titration method, with a precision of ±1 μmol kg^−1^. Chl *a* measurements were made using a Turner 10‐AU fluorometer (Turner Designs, Inc.) (Holm‐Hansen et al., 1965) and were calibrated against a pure Chl *a* standard (Sigma). See more description of sampling and analysis of nutrients, DO, and Chl *a* in Arrigo et al. (2017). DIC and TA samples were collected into 250 mL borosilicate glass bottles poisoned with 100 μL saturated HgCl_2_ solution to prevent biological activity following the procedure described in Dickson et al. (2007). DIC and TA samples were analyzed at the University of Southampton, UK via coulometric (DIC) and potentiometric (TA) titration methods using a VINDTA 3C (Marianda), with a precision of ±2 μmol kg^−1^.

In the summer cruise, nutrient data were measured onboard using a San^++^ automated continuous flow autoanalyzer with a precision of ±2% (SKALAR Inc., Netherlands). The detection limits were 0.1 μM for ${\text{NO}}_{3}^{-}$ and 0.03 μM for ${\text{PO}}_{4}^{3-}$. Chl *a* samples were measured following similar procedures to the spring expedition. DO was measured onboard using the spectrophotometric method based on Winkler titration with a precision of ±1 μmol kg^−1^. See more descriptions of sampling and analysis of nutrients and Chl *a* samples in Zhuang et al. (2020). DIC and TA samples were collected following similar procedures described above, and then were analyzed at the University of Delaware, USA. DIC samples were analyzed using a LiCOR LI 7000 infrared CO_2_ detector coupled to an automated DIC analyzer (Apollo SciTech, USA). TA samples were measured via open‐cell potentiometric titration system (Apollo SciTech, USA). The precision of the DIC and TA measurements was ∼0.1% (∼± 2 μmol kg^−1^; Chen et al., 2015).

In the fall cruise, nutrients were analyzed using QuAAtro system. DO was measured via the spectrophotometric Winkler titration method (Kimoto Electric Co. LTD) following the World Ocean Circulation Experiment Hydrographic Program method (Dickson, 1996). The precision of DO measurement was 0.23 μmol kg^−1^ (Kimoto Electric Co. LTD). Chl *a* was measured using a fluorophotometer (Turner Designs) (Welschmeyer, 1994). Nutrient samples were analyzed following the GO‐SHIP Repeat Hydrography Manual (Hydes et al., 2010) using reference materials for nutrients in seawater (Sato et al., 2010). The precision was 0.08% for ${\text{NO}}_{3}^{-}$ and 0.14% for ${\text{PO}}_{4}^{3-}$. See more descriptions of sampling and analysis of nutrient, DO, and Chl *a* in Nishino et al. (2020). DIC was measured using an automated coulometric analyzer (Nippon ANS, Inc.) with a precision of ±0.7 μmol kg^−1^ and TA samples were measured using a custom spectrophotometric system (Nippon ANS, Inc.) with a precision of ±0.57 μmol kg^−1^. Note that DIC and TA samples collected in all three cruises were calibrated using certified reference materials (CRMs) from Scripps Institution of Oceanography.

In addition, discrete *p*CO_2_ was calculated with surface TA and DIC (<10 m) to increase the data coverage over temporal and spatial scales. The *p*CO_2_ was calculated by the seacarb package in R (Gattuso et al., 2018) with carbonate dissociation constants of Millero et al. (2006), as recommended by Evans et al. (2015). The uncertainty of *p*CO_2_ values computed from TA and DIC is estimated to be ±18 μ atm (RMSE) with a mean systematic difference from the observed *p*CO_2_ of 0.5 μ atm (*N* = 111).

### 2.4 Decomposition of Seasonal *p*CO_2_ Change

To explore the dominant drivers for seasonal *p*CO_2_ change in each water mass, we decomposed the net change of *p*CO_2_ into thermal and non‐thermal effects, assuming that these two effects altered *p*CO_2_ within a time frame much shorter than the residence time of the water mass on the Chukchi shelf. Therefore, the net change in *p*CO_2_ (Δ(*p*CO_2_)) could be calculated as the difference between the initial *p*CO_2_ (*p*CO_2ini_) under the ice in spring (538 ± 68 μatm) and the observed *p*CO_2_ (*p*CO_2ob_) at a given sampling time:

(1) $$\Delta \left(p{\text{CO}}_{2}\right)\;=p{\text{CO}}_{2\text{ob}}-p{\text{CO}}_{2\text{ini}}$$

The potential thermal effect on *p*CO_2_ change (Δ( *p*CO_2_)_T_) was driven by the seasonal variation in SST and was quantified by adjusting the initial *p*CO_2_ to the observed temperature (Takahashi et al., 1993):

(2) $$\Delta {\left(p{\text{CO}}_{2}\right)}_{T}=p{\text{CO}}_{2\text{ini}}\times \exp\left(0.0423\times \;\left(T_{\text{obs}}–T_{\text{spring}}\right)\right)\;-p{\text{CO}}_{2\text{ini}}$$

where T_obs_ is the observed temperature and T_spring_ is the mean temperature observed under the ice in spring (−1.60°C). The non‐thermal effect on *p*CO_2_ change (Δ(*p*CO_2_)_nT_) was mainly driven by the seasonal biological CO_2_ drawdown and ice melt, and was calculated as the difference between the observed total net seasonal change and the thermal effect:

(3) $$\Delta {\left(p{\text{CO}}_{2}\right)}_{\text{nT}}=\Delta \left(p{\text{CO}}_{2}\right)-\Delta {\left(p{\text{CO}}_{2}\right)}_{T}$$

### 2.5 Seasonal CO_2_ Flux Calculation

To avoid calculational bias induced by the overweighted impact of highly dense data point concentrated within a small area, we averaged the data points into daily‐grids of 0.25° latitude × 0.25° longitude, and then we calculated seasonal air‐sea CO_2_ flux for each grid following:

(4) $${\text{FCO}}_{2}=\;K_{s}\cdot k_{{\text{CO}}_{2}}\cdot \Delta p{\text{CO}}_{2}$$

where K_s_ is the solubility of CO_2_ and k_CO2_ is the CO_2_ gas transfer velocity. K_s_ was calculated using underway SST and SSS (Weiss, 1974). The value of k_CO2_ (cm/hr) is estimated from the second moment of wind speed at 10 m above the sea surface, <U_10_^2^> (Wanninkhof, 2014):

(5) $$k_{{\text{CO}}_{2}}=0.251\cdot <{U_{10}}^{2}>\cdot {\left(\text{Sc}/660\right)}^{-0.5}\cdot \left(1\;-\frac{ice\%}{100}\right)$$

To calculate <U_10_^2^>, we used the wind product from the NCEP‐DOE Reanalysis 2 data set (https://www.esrl.noaa.gov/psd/data/gridded/data.ncep.reanalysis2.html). A regional mean of <U_10_^2^> over the study period was derived from the 6‐hr wind speed squared. The term ice% is the sea ice concentration, which is averaged from daily ice% data obtained from the Scanning Multichannel Microwave Radiometer (SMMR) on the Nimbus‐7 satellite and the Special Sensor Microwave/Imager (SSM/I) sensors on the Defense Meteorological Satellite Program's (DMSP)‐F8, ‐F11, and ‐F13 satellites (https://nsidc.org/data/nsidc-0079; Comiso, 2017). For the area with heavy ice covered, we adopted Takahashi et al. (2009) approach that for sea ice where satellite observation is >90% ice cover, a 10% open water area is assumed. In this way, 10% of the open water persisted for CO_2_ flux in the area with >90% ice cover where satellite data with resolution of 25 km × 25 km fails to resolve fine scale structure, such as leads.

The air‐sea CO_2_ gradient ($\Delta p{\text{CO}}_{2}$) is calculated as:

(6) $$\Delta p{\text{CO}}_{2}=p{\text{CO}}_{2}^{\text{sea}}\;–\;p{\text{CO}}_{2}^{\text{air}}$$

where the $p{\text{CO}}_{2}^{\text{sea}}$ is from underway observations and the $p{\text{CO}}_{2}^{\text{air}}$ is derived from monthly average atmospheric CO_2_ concentrations in dry air (xCO_2_ (ppm)) measured at Point Barrow, Alaska (https://www.esrl.noaa.gov/gmd/dv/data/index.php?parameter_name=Carbon%2BDioxide%26frequency=Monthly%2BAverages%26site=BRW; Thoning et al., 2021). Then xCO_2_ is corrected for water vapor pressure content to $p{\text{CO}}_{2}^{\text{air}}$:

(7) $$p{\text{CO}}_{2}^{\text{air}}={\text{xCO}}_{2}\cdot \left(\text{Psl}-\text{Pw}\right)$$

where Psl is sea level pressure and Pw is water vapor pressure. Regional mean Psl over the study period was averaged from a satellite reanalysis product (NCEP‐DOE Reanalysis 2, https://www.esrl.noaa.gov/psd/data/gridded/data.ncep.reanalysis2.html). Pw was calculated from Psl and SST (Buck, 1981).

### 2.6 Estimates of Net Community Production

The NCP can be estimated from the nutrient and DIC changes in the upper mixed layer. Since the residence time of water masses on the Chukchi Sea shelf (3–7.5 months) (Stabeno et al., 2018; Woodgate & Peralta‐Ferriz, 2021) is longer than the timespan of a phytoplankton bloom (<1–2 months), we can assume that water masses move as a single parcel over the shelf, where physical (mostly warming and freshening) and biogeochemical changes occur over time. Therefore, at any particular location, the observed drawdown of nutrients and DIC in the surface mixed layer can be attributed to NCP. However, nutrient and DIC concentrations are not only influenced by NCP, but also by meltwater/river‐runoff dilution, and in the case of DIC, by air‐sea CO_2_ exchange and CaCO_3_ dissolution and formation. Thus, to quantify the biological‐induced changes in nutrients, DIC, and TA, we normalized all nutrient (NO_3_^−^ and ${\text{PO}}_{4}^{3-}$), DIC and TA concentrations to a reference salinity (S_0_, i.e., mean salinity under the ice in spring) to remove the impact of Arctic Ocean freshwater input into the Pacific winter water. As river discharge impact in spring and early summer is either limited to the estuarine and nearshore areas within the Arctic or treated as part of the Pacific water endmember (Text S1 and Figure S1 in Supporting Information S1), the salinity change in Chukchi Sea is mainly attributed to ice melt dilution for period of interest (spring to early summer); thus, non‐zero meltwater endmembers are used (Friis et al., 2003; Jiang et al., 2008):

(8) $$\text{nDIC}=\;\frac{\text{DIC}-{\text{DIC}}_{S=0}}{S}\times S_{0}+{\text{DIC}}_{S=0}$$

(9) $$\text{nTA}=\;\frac{\text{TA}-{\text{TA}}_{S=0}}{S}\times S_{0}{+\text{TA}}_{S=0}$$

(10) $${\text{nNO}}_{3}^{-}=\frac{{\text{NO}}_{3}^{-}-{{\text{NO}}_{3}^{-}}_{\;S=0}}{S}\times S_{0}+{{\text{NO}}_{3}^{-}}_{\;S=0}$$

The meltwater salinity, DIC and TA values are set as 5, 400 μmol kg^−1^, 460 μmol kg^−1^ (Rysgaard et al., 2007), respectively, which are equivalent to DIC_S=0_ = 60 μmol kg^−1^ and TA_S=0_ = 106 μmol kg^−1^ (Cai et al., 2010; Ouyang et al., 2020). The contributions of meltwater to ${\text{NO}}_{3}^{-}$ (μM) and ${\text{PO}}_{4}^{3-}$ (μM) concentrations are negligible (Clark et al., 2020).

The deficit of n${\text{NO}}_{3}^{-}$ (or n${\text{PO}}_{4}^{3-}$) is the result of phytoplankton uptake between the time before the phytoplankton bloom begins (t_1_) in spring and the time of sampling (t_2_). Then, the vertical integral of the deficit of n${\text{NO}}_{3}^{-}$ (or n${\text{PO}}_{4}^{3-}$) within surface mixed layer is assumed to be equal to NCP at any given location over the time interval between t_1_ and t_2_ (Δt),

(11) $${\text{NCP}}_{N‐\text{based}}=\left({{\mathrm{nNO}}_{3}^{-}}_{t1}\;-\;{{\mathrm{nNO}}_{3}^{-}}_{t2}\right)\;\text{MLD}\;/\mathrm{\Delta t}$$

Analogue equations of Equations 10 and 11 can be written for n${\text{PO}}_{4}^{3-}$. The average mixed layer depth (MLD) of 25 m was used in this study. While this is a highly simplified treatment, the MLD uncertainty would apply equally to C‐, N‐, and P‐based NCP. The estimates of nutrient‐based NCP were then converted to carbon units by multiplying by a M C:N:P uptake ratio of 106:16:1 (Arrigo et al., 2017; Codispoti et al., 2013; Hansell et al., 1993; Redfield, 1958).

The nDIC change reflects the influence of both phytoplankton uptake, air‐sea CO_2_ exchange, and CaCO_3_ dissolution and formation. Therefore, we calculated biological‐induced nDIC change by subtracting the DIC added by the CO_2_ influx and possible CaCO_3_ formation and dissolution (CaCO_3_ + H_2_O + CO_2_ ↔ Ca^2+^+ 2HCO_3_^−^):

(12) $${\text{NCP}}_{\text{DIC}‐\text{based}}=\left({\text{nDIC}}_{t1}\times \rho_{t1}-{\text{nDIC}}_{t2}\times \rho_{t2}-\Delta {\text{DIC}}_{{\text{CaCO}}_{3}}\right)\times \;\mathrm{MLD}/\Delta t-{\text{CO}}_{2}\;\text{flux}$$

where ρ is the water density and CO_2_ flux term was calculated following Equation 4. The term of $\Delta {\text{DIC}}_{\text{CaCO}3}$ can be quantified as half of the change in salinity‐normalized TA (nTA) corrected for the removal of NO_3_^−^ to form organic matter (Brewer & Goldman, 1976; Lee, 2001):

(13) $$\Delta {\text{DIC}}_{{\text{CaCO}}_{3}}=\;\left(\Delta \text{nTA}\times \rho -\Delta {{\mathrm{NO}}_{3}}^{-}\right)\times \;0.5$$

Note that due to lack of information we are not able to differentiate biological calcification and the dissolution of CaCO_3_ mineral (ikaite) trapped in the melting ice.

### 2.7 Box Model

The goal of the box model is to investigate how a flexible C:N uptake ratio by phytoplankton affects DIC fixation and air‐sea CO_2_ fluxes in the Chukchi Sea. Similar box model approaches have been described in previous studies (Ouyang et al., 2020, 2021). Briefly, the initial TA and DIC values were set to the mean value of observations in the surface mixed layer in late spring, and the initial sea surface *p*CO_2_ was calculated from the initial TA and DIC using the seacarb package in R (Gattuso et al., 2018). TA in the subsequent season was linearly interpolated based on observations. For each simulation step, sea surface *p*CO_2_ was calculated from TA and DIC at the corresponding step. The change in DIC inventory (∆DIC) in the surface mixed layer was calculated as

(14) $$\Delta {\text{DIC}}_{t}=\;\left({\text{FCO}}_{2t}+{\text{NCP}}_{t}\right)\times \tau /\left(\text{MLD}\times \rho \right)\;+\Delta {\text{DIC}}_{\left(\text{diluted}\right)t}$$

where FCO_2*t*_, NCP_*t*_, and ∆DIC_(diluted)*t*_ indicate the changes in DIC inventory in the mixed layer induced by the CO_2_ air‐sea flux, NCP, and dilution by meltwater at simulation time step *t*, respectively. In this case, the simulation time interval (τ) is 1 day. The surface seawater density, ρ, was calculated using SST and SSS. The FCO_2_ term was calculated with Equation 4. The ∆DIC_(diluted)*t*_ term was computed by simplifying the ice melt dilution and assuming that the ratio of TA/DIC in the thin first‐year ice nearly equals that in the surface seawater (Rysgaard et al., 2007). Thus,

(15) $$\Delta {\text{DIC}}_{\left(\text{diluted}\right)t}=\left({\text{TA}}_{t+1}-{\text{TA}}_{t}\right)/{\text{TA}}_{t}\times {\text{DIC}}_{t}$$

Because the Chukchi Sea is N‐limited during the growing season (Mills et al., 2015, 2018), NCP_t_ was calculated based on nitrate; NCP_N‐based_. Two scenarios with different C:N uptake ratios were examined in the box model. For the fixed‐stoichiometry scenario, a C:N uptake ratio of 6.6 (Redfield ratio) was used. For the non‐Redfield scenario, much higher C:N uptake ratios were used (i.e., 10.9 and 12.1 in Table 5). Therefore,

(16) $${\text{NCP}}_{t}={\text{NCP}}_{N‐\text{based}}\times \text{C:N}\;\text{ratio}$$

**Table 3:** Seasonal Variations in Biogeochemical Properties in the Water Column

Periods	Layers	Temperature (°C)		Salinity		TA (μmol kg^−1^)		DIC (μmol kg^−1^)		AOU (μmol kg^−1^)		Chl *a* (μg l^−1^)		NO_3_^−^ (μM)		PO_4_^3−^ (μM)	
		Southern Chukchi	Northern Chukchi	Southern Chukchi	Northern Chukchi	Southern Chukchi	Northern Chukchi	Southern Chukchi	Northern Chukchi	Southern Chukchi	Northern Chukchi	Southern Chukchi	Northern Chukchi	Southern Chukchi	Northern Chukchi	Southern Chukchi	Northern Chukchi
Early spring	Surface	−1.28 ± 0.19	−1.71 ± 0.04	31.9 ± 0.4	32.1 ± 0.6	2,205 ± 7	2,225 ± 28	2,068 ± 35	2,173 ± 41	−13.9 ± 3.5	35.6 ± 23.2	4.4 ± 3.1	0.9 ± 1.7	9.57 ± 5.29	10.54 ± 3.95	1.33 ± 0.30	1.66 ± 0.32
	Bottom	−1.03 ± 0.15	−1.74 ± 0.04	32.2 ± 0.3	32.4 ± 0.4	2,212 ± 28	2,236 ± 18	2,112 ± 70	2,191 ± 27	−27.0 ± 25.8	49.4 ± 20.2	2.4 ± 3.2	0.5 ± 1.2	10.3 ± 3.30	12.18 ± 3.11	1.41 ± 0.27	1.82 ± 0.27
Late spring	Surface	NA	−1.61 ± 0.36	NA	32.2 ± 0.5	NA	2,229 ± 23	NA	2,153 ± 47	NA	14.3 ± 38.0	NA	2.8 ± 2.2	NA	10.57 ± 4.33	NA	1.59 ± 0.35
	Bottom	NA	−1.73 ± 0.04	NA	32.6 ± 0.2	NA	2,243 ± 13	NA	2,188 ± 23	NA	40.9 ± 30.1	NA	2.9 ± 3.1	NA	13.23 ± 2.81	NA	1.89 ± 0.26
Early Summer	Surface	5.95 ± 1.80	3.76 ± 2.79	31.4 ± 1.0	30.7 ± 0.9	2,187 ± 40	2,154 ± 37	1,940 ± 49	1,954 ± 51	−44.6 ± 42.0	−8.2 ± 59.5	1.4 ± 1.2	1.8 ± 3.2	0.27 ± 0.20	0.62 ± 1.51	0.46 ± 0.11	0.62 ± 0.20
	Bottom	3.93 ± 1.61	−0.42 ± 0.99	31.9 ± 0.6	32.1 ± 0.4	2,204 ± 31	2,225 ± 25	2,215 ± 64	2,067 ± 78	4.3 ± 34.6	39.9 ± 54.6	2.6 ± 4.0	2.6 ± 2.4	3.67 ± 4.50	4.96 ± 8.29	0.84 ± 0.34	1.07 ± 0.55
Late Summer	Surface	4.46 ± 1.55	NA	32.1 ± 0.3	NA	2,199 ± 20	NA	2,012 ± 61	NA	−16.6 ± 17.1	NA	18.8 ± 10.0	NA	3.95 ± 3.46	NA	0.75 ± 0.35	NA
	Bottom	2.27 ± 0.27	NA	32.5 ± 0.2	NA	2,211 ± 12	NA	2,161 ± 24	NA	88.5 ± 13.7	NA	2.3 ± 0.7	NA	18.01 ± 2.53	NA	2.08 ± 0.25	NA
Fall	Surface	5.33 ± 0.99	3.10 ± 0.58	31.6 ± 0.2	31.1 ± 0.2	2,180 ± 14	2,249 ± 12	2,008 ± 27	1,971 ± 13	−5.0 ± 4.8	−4.4 ± 11.4	5.8 ± 2.9	0.7 ± 0.4	2.24 ± 1.07	0.06 ± 0.03	0.62 ± 0.12	0.45 ± 0.03
	Bottom	3.15 ± 0.37	0.66 ± 1.56	32.4 ± 0.4	32.2 ± 0.3	2,207 ± 21	2,208 ± 15	2,111 ± 45	2,105 ± 59	44.7 ± 32.0	35.1 ± 48.7	2.2 ± 1.1	0.6 ± 0.3	11.53 ± 4.48	4.60 ± 2.51	1.56 ± 0.35	1.34 ± 0.41

*Note.* Mean and standard deviation are reported for both the surface mixed layer and the bottom layer. No observations were made in the southern Chukchi Sea in late spring and in northern Chukchi Sea in late summer.

**Table 4:** Seasonal Variations in CO_2_ Flux (mmol m^−2^ d^−1^) in the Chukchi Sea

	Southern Chukchi sea (<69.5°N)			Northern Chukchi sea (>69.5°N)		
Periods	Regional mean	ACW	Non‐ACW	Regional mean	ACW	Non‐ACW
Spring	−2.5 ± 1.5 (n = 15)	NA	NA	1.1 ± 1.1 (n = 145)	NA	−2.2 ± 2.4 (n = 6)
Early Summer	−15.9 ± 6.0 (n = 73)	−14.8 ± 5.7 (n = 62)	−22.3 ± 1.9 (n = 11)^c^	−7.2 ± 3.4 (n = 377)	−8.8 ± 2.6 (n = 58)	−7.0 ± 3.4 (n = 319)^c^
Late Summer	−18.3 ± 8.9 (n = 75)	−16.7 ± 8.1 (n = 59)	−24.3 ± 9.6 (n = 16)^b^	−13.3 ± 6.6 (n = 117)	−12.5 ± 5.5 (n = 54)	−14.2 ± 7.4 (n‐63)
Fall	−10.5 ± 8.4 (n = 17)	−13.7 ± 4.4 (n = 14)	4.4 ± 6.1 (n = 3)^a^	−21.1 ± 2.9 (n = 47)	−19.8 ± 1.8 (n = 15)	−21.7 ± 3.2 (n = 32)^a^

*Note*. We estimated CO_2_ fluxes separately in two types of water masses: the ACW (including RW) and non‐ACW (including BSW and MW but excluding NVWW and RWW) for each season. Footnotes indicates if the mean of CO_2_ flux in the non‐ACW is statistically different from that in the ACW (t‐test).

^a^ *p* < 0.05.

^b^ *p* < 0.01.

^c^ *p* < 0.001.

**Table 5:** Net Community Production Estimates in the Surface Mixed Layer Based on nDIC, ${\text{nNO}}_{3}^{-}$ and ${\text{nPO}}_{4}^{3-}$ Changes for the Period Between the Spring and the Early Summer of 2014 in the Chukchi Sea

Location	Δ (nTA)	Δ (nDIC)	Δ ($n{\mathrm{NO}}_{3}^{-}$)	Δ ($n{\mathrm{PO}}_{4}^{3-}$)	Growing season	N	NCP‐nDIC	NCP‐${\text{nNO}}_{3}^{-}$		NCP‐${\mathrm{nPO}}_{4}^{3-}$		C:N uptake ratio	C:P uptake ratio
	μmol kg^−1^	μmol kg^−1^	μM	μM	d		mmol C m^−2^ d^−1^	mmol N m^−2^ d^−1^	mmol C m^−2^ d^−1^	mmol P m^−2^ d^−1^	mmol C m^−2^ d^−1^		
Southern	−34 ± 37	79 ± 87	10.24 ± 0.20	1.19 ± 0.11	73	18	38.4 ± 26.2	3.51 ± 0.07	23.2 ± 0.5^a^	0.41 ± 0.04	43.3 ± 4.0	10.9	94.0
Northern	−36 ± 34	95 ± 51	9.92 ± 1.57	0.94 ± 0.21	49	13	61.4 ± 20.0	5.06 ± 0.80	33.4 ± 5.3^b^	0.48 ± 0.11	50.9 ± 11.3	12.1	128.0

*Note*. Differences in nTA, nDIC, ${\text{nNO}}_{3}^{-}$ and ${\text{nPO}}_{4}^{3-}$ were calculated as Variable_spring_ – Variable _early summer_, thus negative values indicate an increase in variable in the later season. Nutrient‐based NCP were converted to carbon (C) units assuming a Redfield M C:N:P uptake ratio of 106:16:1. C:N and C:P uptake ratios are defined as NCP_nDIC_: NCP‐${\text{nNO}}_{3}^{-}$ and NCP‐_nDIC_: NCP‐nPO_4_^3−^. Footnotes indicates if the mean of NCP‐${\text{nNO}}_{3}^{-}$ and NCP‐nPO4^3−^ in unit of mmol C m^−2^ d^−1^ are statistically different than that in NCP nDIC (t‐test).

^a^ *p* < 0.05.

^b^ *p* < 0.01.

And DIC at time step t + 1 was iteratively calculated as follows:

(17) $${\text{DIC}}_{t+1}={\text{DIC}}_{t}+\Delta {\text{DIC}}_{t}$$

With the new DIC and TA, a new *p*CO_2_ was calculated for the next simulation step, and this simulation process repeated until the last day of the simulation.

## 3 Results

### 3.1 Sea Surface Temperature, Salinity, and *p*CO_2_

Underway SST, SSS, and *p*CO_2_ were measured during five cruises in the Chukchi Sea, from May through September in 2014, providing a consistent way to examine the seasonal transition of the ocean from a CO_2_ source to a sink and to explore the role of changing water masses.

**Figure 2:**
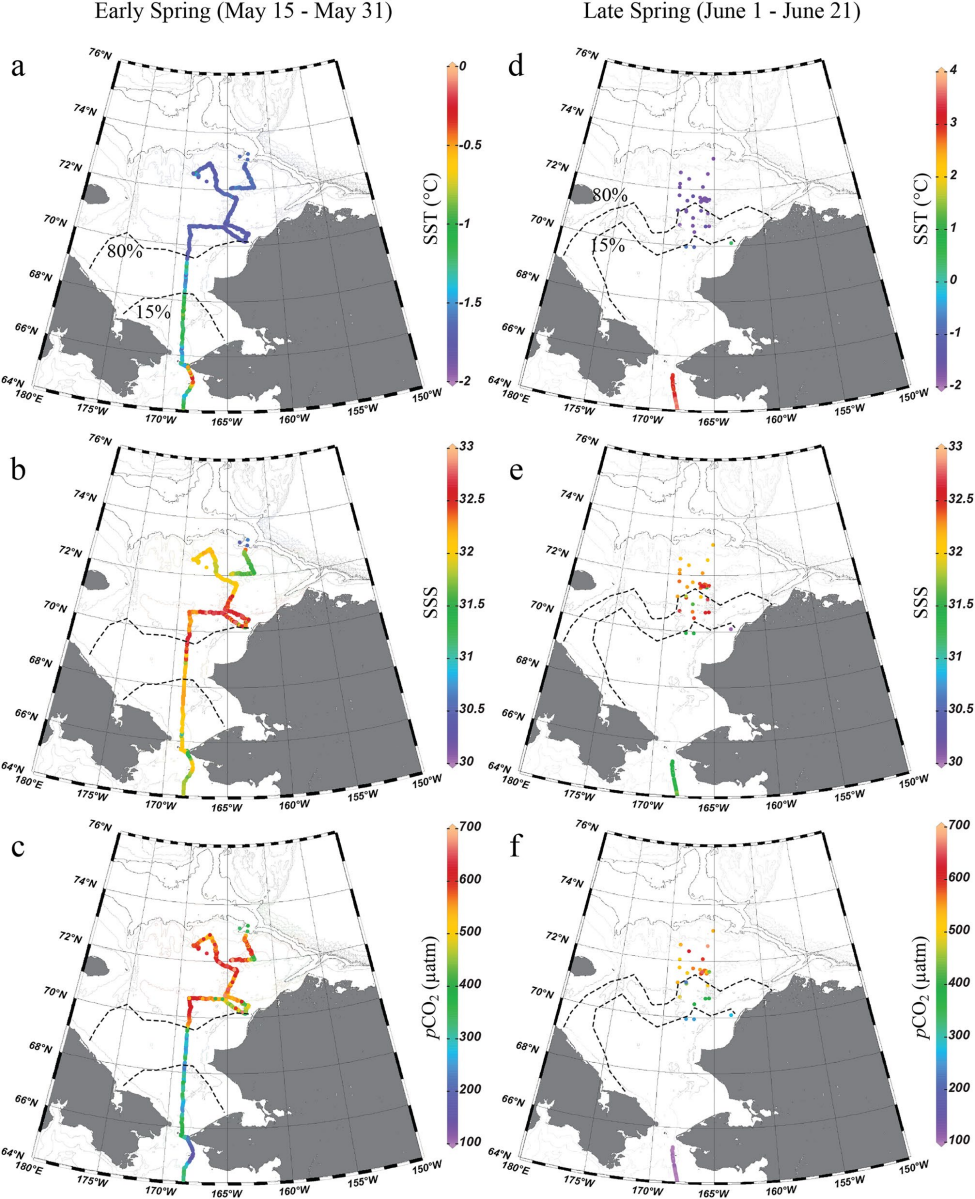
Sea surface temperature, salinity, and *p*CO_2_ in the spring of 2014 in the Chukchi Sea. The observations are presented in two periods, the early spring (15–31 May) and the late spring (1–21 June). The dashed lines indicate the sea ice concentration contours of 15% and 80% on the last day of each period, 31 May (a–c) and 21 June (d–f), showing the possible maximum ice retreat for each period (National Snow and Ice Data Center, Fetterer et al., 2017, https://doi.org/10.7265/N5K072F8). Figures were created using Ocean Data View (Schlitzer, 2016).

Because sea ice melt began in the southern portion of the Chukchi Sea in early June, we divided the spring observations into two periods: early spring (15–31 May) and late spring (1–21 June 1). Clearly, SST distribution was greatly affected by sea ice (Figure 2a). SST was near freezing (−1.67 ± 0.07°C) under the ice in the north, while SST in the south was slightly warmer (−1.28 ± 0.28°C). The location of SST fronts corresponded well with the edge of heavy ice cover around 69.5°N (ice concentration ∼80%). However, the distribution of SSS was not as closely correlated with ice as SST was (Figure 2b). In the southern portion of Chukchi Sea, the surface water was relatively fresher due to mixing with newly melted water and river discharge. The saltier water appeared in the eastern and central Chukchi Sea under the ice. Interestingly, relatively warmer and fresher water was also found in the northeast part of Chukchi Sea toward the shelfbreak (Figures 2a and 2b), which was likely due to local ice melt events. The average *p*CO_2_ in the south was 309 ± 50 μatm, which was much lower than that in the north (536 ± 74 μatm). Such a contrasting pattern in *p*CO_2_ indicated that the southern ice‐free Chukchi Sea had already become a CO_2_ sink while the northern ice‐covered Chukchi Sea still remained supersaturated with respect to atmospheric CO_2_ (∼400 μatm) and was a potentially strong CO_2_ source in early spring (Figure 2c).

As sea ice retreated northwards in the late spring (Figures 2d–2f), more warm and fresh waters advected from south to north. However, their impacts were confined by sea ice. The SST, SSS, and *p*CO_2_ values in the ice‐covered area were similar to those earlier in the spring. Only a few observations south of the ice edge showed signals of warmer and fresher Pacific summer waters. Although there were no observations in the southern Chukchi Sea during this period, we expect that water there might have become warmer and fresher with much lower *p*CO_2_ compared to early spring, which was confirmed by observations on the last day (21 June) of the spring cruise in the south of Bering Strait (Figures 2d–2f).

In early summer (9 July–7 August), the ice edge retreated further northwards to around 71–72°N (Figures 3a–3f). The mean SST increased to 6.64 ± 1.22°C in the southern and central Chukchi Sea but remained low (−0.83 ± 1.31°C) under the ice in the northern Chukchi Sea (Figures 3a and 3d). The distribution of SSS was not only affected by the northward advection of water but also by dilution by meltwater. Compared to spring, the mean SSS (31.0 ± 0.7) over much of the southern and central Chukchi Sea had decreased by 0.5–1.0, although this decrease was more substantial (by 3–4) near the shelfbreak (Figures 3b and 3e). The most notable change in the carbonate system between spring and early summer was a widespread reduction in sea surface *p*CO_2_. The lowest *p*CO_2_ values were found in the vicinity of the Bering Strait in the south and along the ice edge in the northern Chukchi Sea (Figures 3c and 3f).

Figure 3Sea surface temperature, salinity, and *p*CO_2_ in the summer of 2014 in the Chukchi Sea. The observations are presented in four periods depending on the timing of cruises, 9–26 July (a–c), 27 July–7 August (d–f), 12–24 August (g–i) and 3–9 September (j–l). The dashed lines indicate the 15% sea ice concentration contour on the last day of each period, 26 July (a–c), 7 August (d–f), 24 August (g–i) and 9 September (j–l), showing the possible maximum ice retreat for each period (National Snow and Ice Data Center, Fetterer et al., 2017, https://doi.org/10.7265/N5K072F8). Figures were created using Ocean Data View (Schlitzer, 2016).

**Figure:**
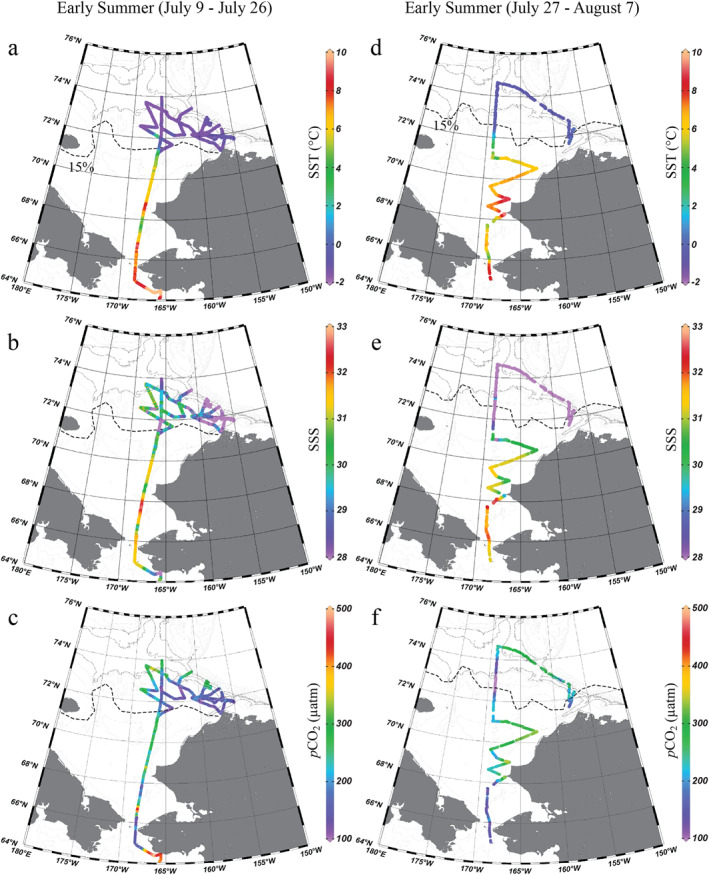

**Figure:**
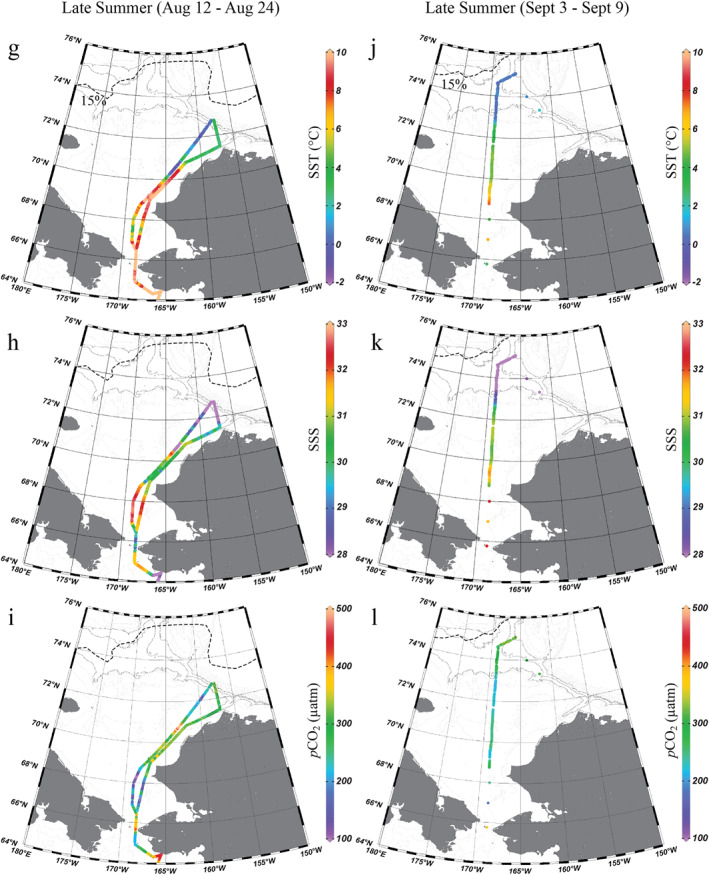

The Chukchi Sea had become a completely ice‐free region in late summer (12 August 12–9 September 9; Figures 3g–3l). SST continued to increase over the entire Chukchi shelf (Figures 3g and 3j). The highest SST appeared in the middle of August along the Alaska coast, reaching up to ∼11°C (Figure 3g). The SSS showed a similar distribution as in early summer, with saltier water in the southern and central Chukchi Sea and fresher water in the eastern and northern portion as a result of massive sea‐ice melt and river discharge (Figures 3h and 3k). Compared to early summer, the mean sea surface *p*CO_2_ in late summer increased from 202 ± 63 μatm to 265 ± 69 μatm but remained much lower than the atmospheric *p*CO_2_ (Figures 3i and 3l). It is noteworthy that the lowest *p*CO_2_ was observed repeatedly north of Bering Strait and in the area surrounding Hanna Shoal (Figures 3i and 3l).

**Figure 4:**
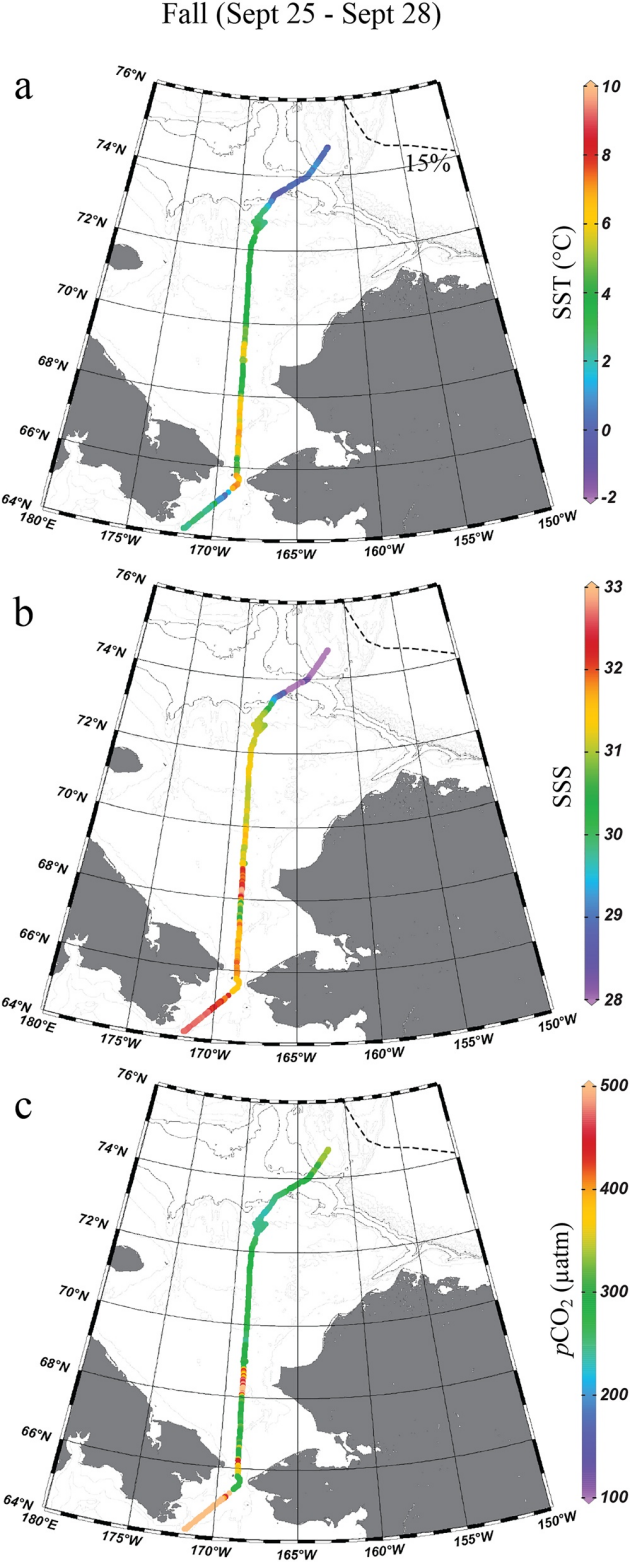
Sea surface temperature, salinity, and *p*CO_2_ in the fall of 2014 (25–28 September) in the Chukchi Sea. The dashed lines indicate the 15% sea ice concentration contour on the last day of survey, 28 September (j–l), showing the possible maximum ice retreat for each period (National Snow and Ice Data Center, Fetterer et al., 2017, https://doi.org/10.7265/N5K072F8). Figures were created using Ocean Data View (Schlitzer, 2016).

Between late summer and fall, the mean SST over the entire shelf decreased from 5.42 ± 3.04°C to 3.49 ± 1.73°C, while the mean SSS remained nearly unchanged (Figures 4a and 4b). For most of the Chukchi Sea, surface water *p*CO_2_ was lower than the atmospheric *p*CO_2_, averaging 283 ± 50 μatm, indicating that the CO_2_ sink lasted into fall. However, extremely high *p*CO_2_ started to appear in the northern Bering Sea along the western pathway of Pacific Summer Water (Figure 4c).

### 3.2 Water Column Structure

Discrete samples of biogeochemical parameters were collected during three cruises (Table 1). Since these three cruises conducted survey sections along the Central Channel, we are able to show the seasonal evolution of biogeochemical properties in the water column along a transect from the Bering Strait extending to the shelf break in the northern Chukchi Sea.

**Figure 5:**
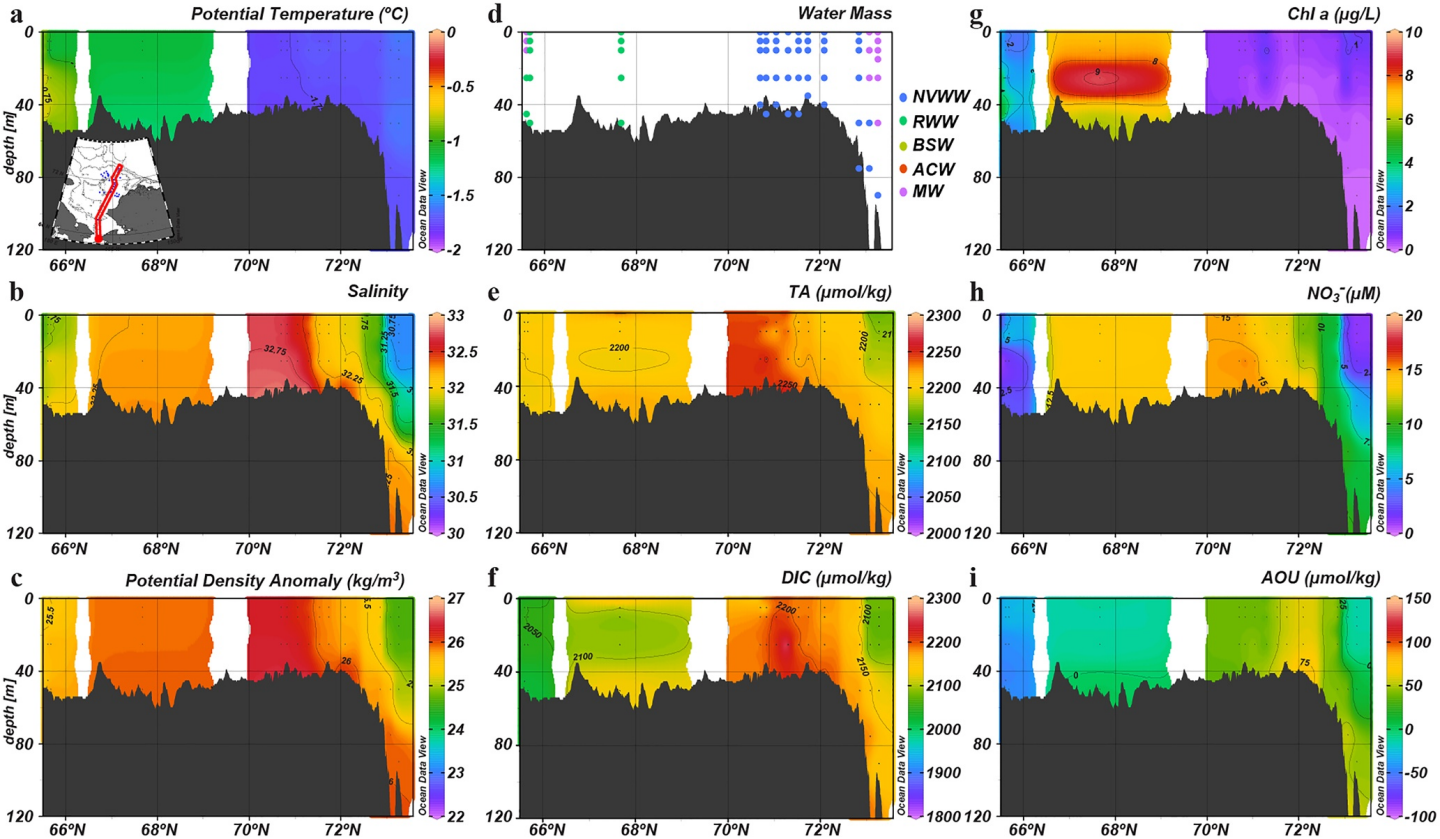
Vertical distributions of physical and biogeochemical parameters in the early spring (15–31 May) along the central Chukchi Sea. (a) Potential temperature, (b) salinity, (c) potential density anomaly, (d) water mass, (e) TA, (f) dissolved inorganic carbon, (g) chlorophyll *a*, (h) ${\text{NO}}_{3}^{-}$ and (i) apparent oxygen utilization.

In early spring, water columns on the Chukchi shelf were well mixed, exhibiting little vertical structure for most biogeochemical properties (Figure 5). The exceptions were a subsurface (20–30 m) Chl *a* maximum in the southern Chukchi Sea (Figure 5g) and the frontal structures of large gradient in DIC and ${\text{NO}}_{3}^{-}$ across the shelf breaks (73°N; Figures 5f and 5h). However, latitudinal patterns were significantly different from the south to the north, which was due to different levels of primary production regulated by the timing of ice melting. Seawater temperature and salinity under the ice were within a narrow range of −1.78°C to −1.60°C and 32 to 33, respectively, indicating that during this period, almost all waters on the northern shelf were NVWW (Figure 5d). Low concentrations of Chl *a* (<1 μg l^−1^), high ${\text{NO}}_{3}^{-}$ (>15 μM), and positive apparent oxygen utilization (AOU) beneath the ice suggested that the spring bloom had not yet started there (Figures 5g–5i) and the ambient high DIC was likely accumulated from remineralization in the previous winter water (Figure 5f). In contrast, primary production within RWW had begun in the southern Chukchi Sea, indicated by a much higher Chl *a* concentration (>4 μg l^−1^), lower ${\text{NO}}_{3}^{-}$, and negative AOU (Figures 5g–5i). As a result, mean DIC in the south was ∼100 μmol kg^−1^ lower than that in the north (Figure 5f and Table 3).

**Figure 6:**
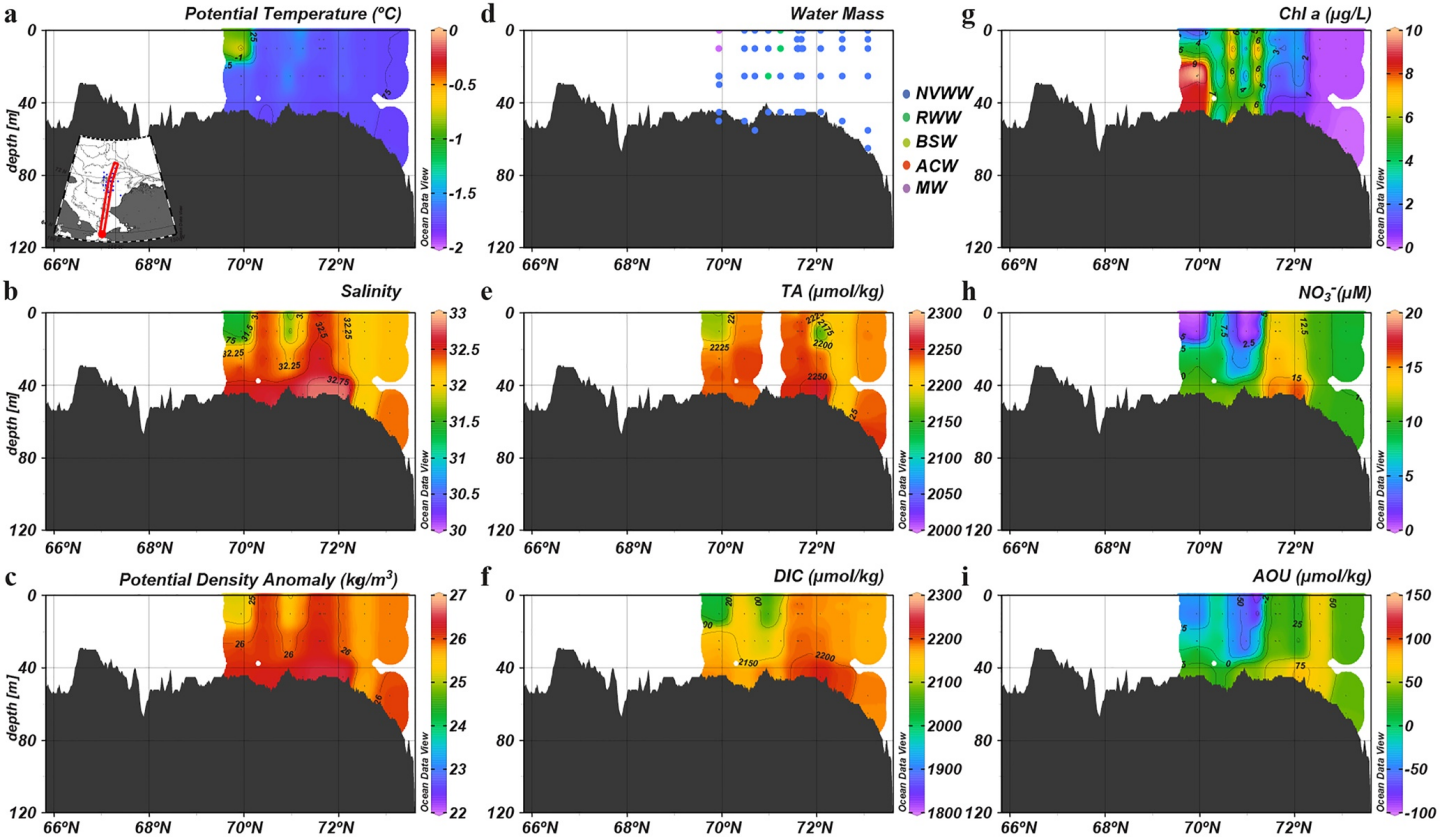
Vertical distributions of physical and biogeochemical parameters in the late spring (1–21 June) along the central Chukchi Sea. (a) Potential temperature, (b) salinity, (c) potential density anomaly, (d) water mass, (e) TA, (f) dissolved inorganic carbon, (g) chlorophyll *a*, (h) ${\text{NO}}_{3}^{-}$ and (i) apparent oxygen utilization.

A few weeks later, as the ice conditions changed, the water at the ice edge became warmer and fresher as a result of local sea ice melt freshening together with atmospheric heating (70.5°N; Figures 6a and 6b). Although most water columns still consisted of NVWW and were well mixed, a two‐layer structure appeared in the southernmost stations (Figure 6c), indicating that seasonal stratification of the water column had begun. In addition, the Chl *a* concentration increased to 2.8 ± 2.2 μg l^−1^ and the previously positive AOU had turned negative, indicating net oxygen production by a phytoplankton bloom (Figure 6i). The late‐spring ice retreat further strengthened primary production in the northern Chukchi Sea. In turn, the associated biological CO_2_ drawdown resulted in lower DIC in the surface layer (Figure 6f).

**Figure 7:**
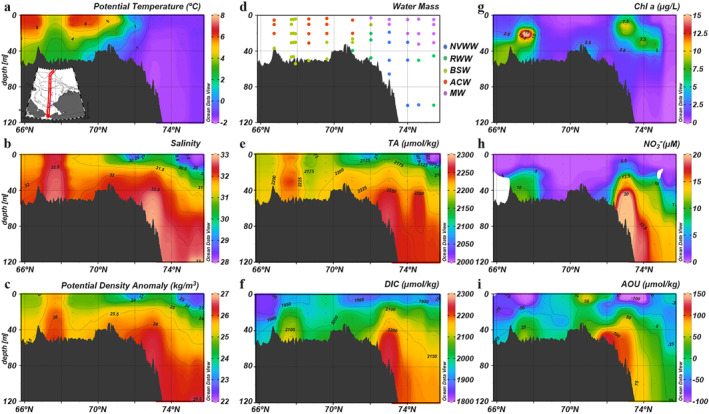
Vertical distributions of physical and biogeochemical parameters in the early summer (27 July 27–7 August) along the central Chukchi Sea. (a) Potential temperature, (b) salinity, (c) potential density anomaly, (d) water mass, (e) TA, (f) dissolved inorganic carbon, (g) chlorophyll *a*, (h) ${\text{NO}}_{3}^{-}$ and (i) apparent oxygen utilization. Note that the color scale of chlorophyll a concentration is different from that of Figures 5, 6 and 9h.

Conditions during the early summer transect were vastly different from the spring conditions (Figure 7). The fully mixed water column representative of spring conditions had transformed into a clear two‐layer structure with a shallow, warm and fresh surface mixed layer with ACW properties separated by a halocline from BSW and winter water near the bottom (Figures 7a and 7d). Strong primary production over the shelf led to a large reduction in DIC and ${\text{NO}}_{3}^{-}$ in the surface mixed layer (Figures 7f and 7h; Table 3). Simultaneously, the surface extremely high O_2_ (AOU < −100 μmol kg^−1^) coincided with the subsurface Chl *a* maximum in the north of Bering Strait and over the shelfbreak (Figures 7g and 7i). Interestingly, the surface extremely low ${\text{NO}}_{3}^{-}$ (<1 μM) and supersaturated DO extended throughout the water column in the central Chukchi Sea (68°N −70°N Figures 7h and 7i), which were likely induced by local mixing events occasionally breaking the two‐layer structure or a mechanical overturning induced by internal wave mixing (Kawaguchi et al., 2015; Nishino et al., 2015).

**Figure 8:**
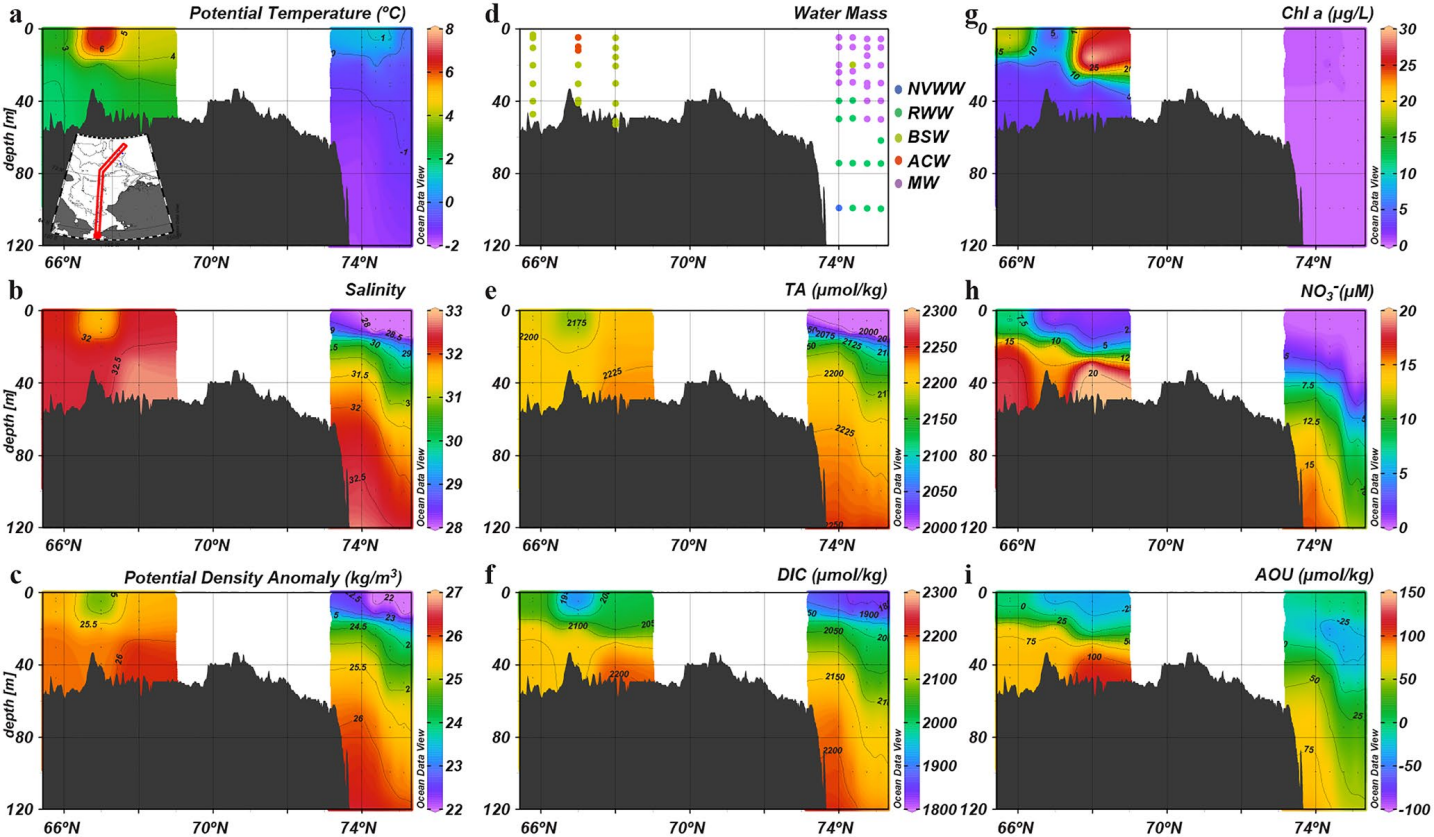
Vertical distributions of physical and biogeochemical parameters in the late summer (3–9 September) along the central Chukchi Sea. (a) Potential temperature, (b) salinity, (c) potential density anomaly, (d) water mass, (e) TA, (f) dissolved inorganic carbon, (g) chlorophyll *a*, (h) ${\text{NO}}_{3}^{-}$ and (i) apparent oxygen utilization. Note that the color scale of chlorophyll a concentration is different from that of Figures 5, 6 and 9h.

Although we only have observations in the southern Chukchi Sea and in the Canada Basin (i.e., no data in the central and northern shelf) in late summer, it is likely that the strong two‐layer stratification remained in the water column over most of the Chukchi Sea (Figure 8). The highest Chl *a* concentration (>25 μg l^−1^) repeatedly appeared at the similar location in the north of Bering Strait associated with BSW (68°N; Figures 8d and 8g) but AOU increased from <−100 to ∼ −30 μmol kg^−1^ (Figure 8i). The surface DIC in the southern Chukchi Sea (probably in the northern Chukchi Sea as well) became higher than in early summer by ∼100 μmol kg^−1^ (Figure 8f). As the water column stratification strengthened, the gradients in DIC, ${\text{NO}}_{3}^{-}$ and AOU between surface and bottom layers became larger (Figures 8f, 8h and 8i; Table 3). The highest ${\text{NO}}_{3}^{-}$ (>20 μM) was observed in the bottom waters in the southern Chukchi Sea.

**Figure 9:**
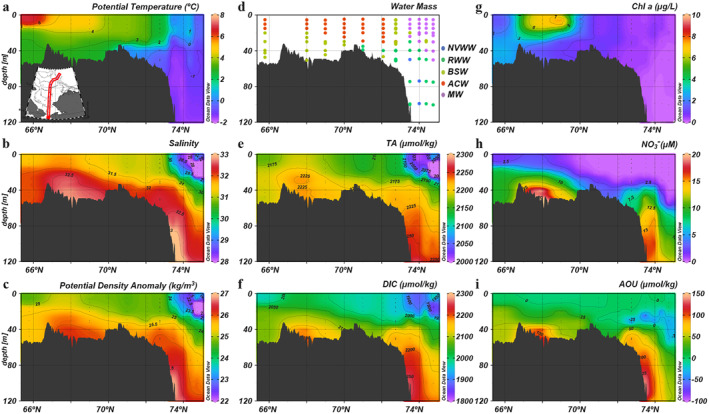
Vertical distributions of physical and biogeochemical parameters in the fall (24–28 September) along the central Chukchi Sea. (a) Potential temperature, (b) salinity, (c) potential density anomaly, (d) water mass, (e) TA, (f) dissolved inorganic carbon, (g) chlorophyll *a*, (h) ${\text{NO}}_{3}^{-}$ and (i) apparent oxygen utilization.

Three weeks later in fall, the stratified vertical structure persisted over most of the shelf area (Figures 9a–9d). However, a widespread reduction in Chl a concentrations suggested that primary production had become much weaker. High Chl a concentrations were only observed in the biological hot spot in the southern Chukchi Sea (68°N; Figure 9g), which was present throughout the entire growing season. ${\text{NO}}_{3}^{-}$ became depleted in the surface waters (Figure 9h), limiting further phytoplankton growth, while replenishment of ${\text{NO}}_{3}^{-}$ was observed in bottom waters in the southern Chukchi Sea. Consequently, surface O_2_ approached equilibrium with the atmosphere (AOU was ∼0 μmol kg^−1^) and surface DIC remained the same as it was in late summer (Figures 9f and 9i).

## 4 Discussion

### 4.1 Water Mass Evolution and CO_2_ Flux

**Figure 10:**
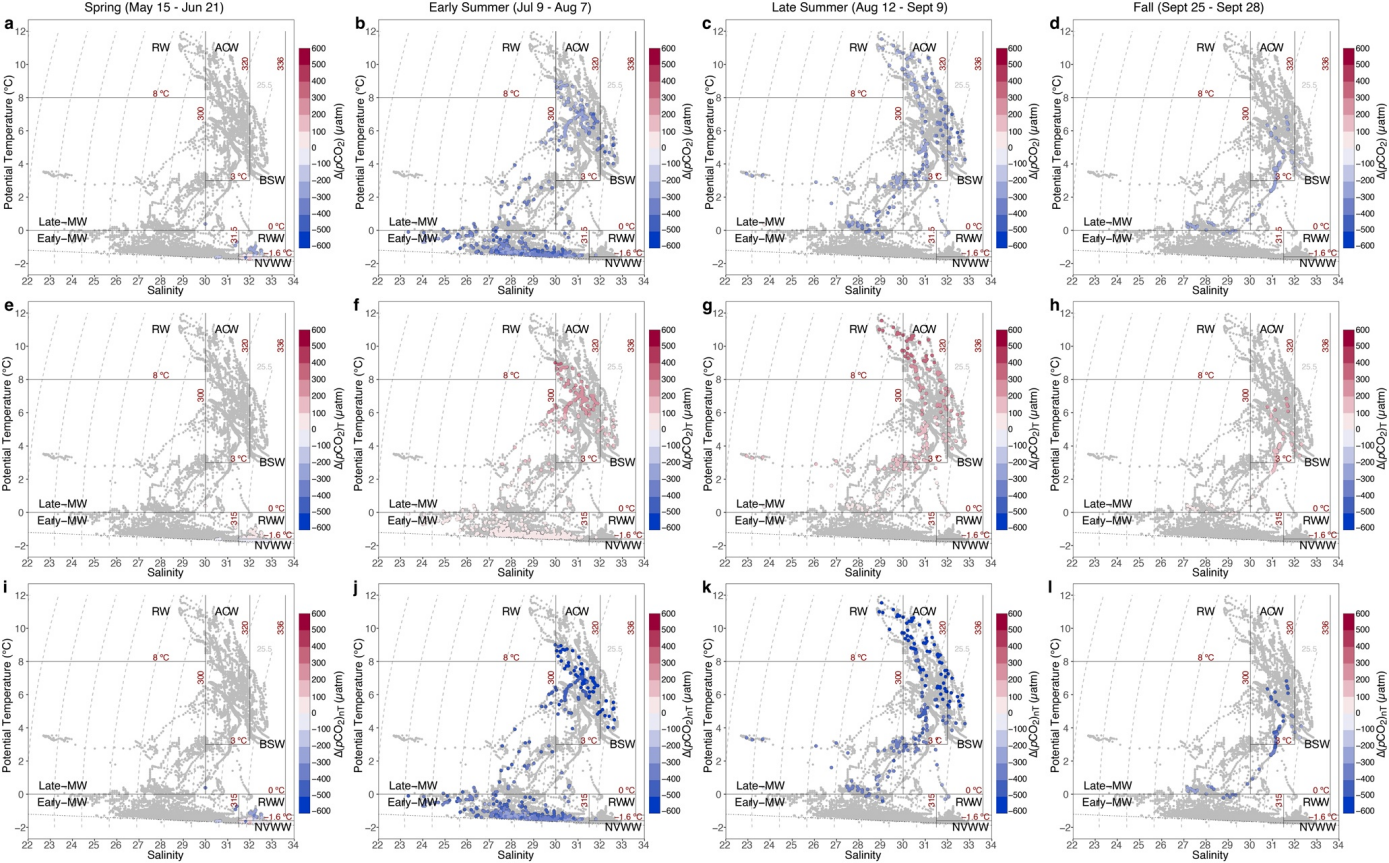
T/S diagram for sea surface *p*CO_2_ in the Chukchi Sea in different seasons. The gray dots represent all underway measurements and colored dots denote the gridded *p*CO_2_ (0.25° latitude × 0.25° longitude) (a–d) *p*CO_2_ difference between initial *p*CO_2_ under the ice (i.e., 538 μatm) and observed *p*CO_2_ (e–g) potential *p*CO_2_ changes induced by thermal effects, and (i–l) *p*CO_2_ changes induced by non‐thermal effects. The approximate water mass boundaries are denoted by solid lines. NVWW is newly‐ventilated winter water; RWW is remnant winter water; BSW is Bering summer water; ACW is Alaskan coastal water; MW is meltwater; RW is river water. The freezing line is indicated by the dashed line.

**Figure 11:**
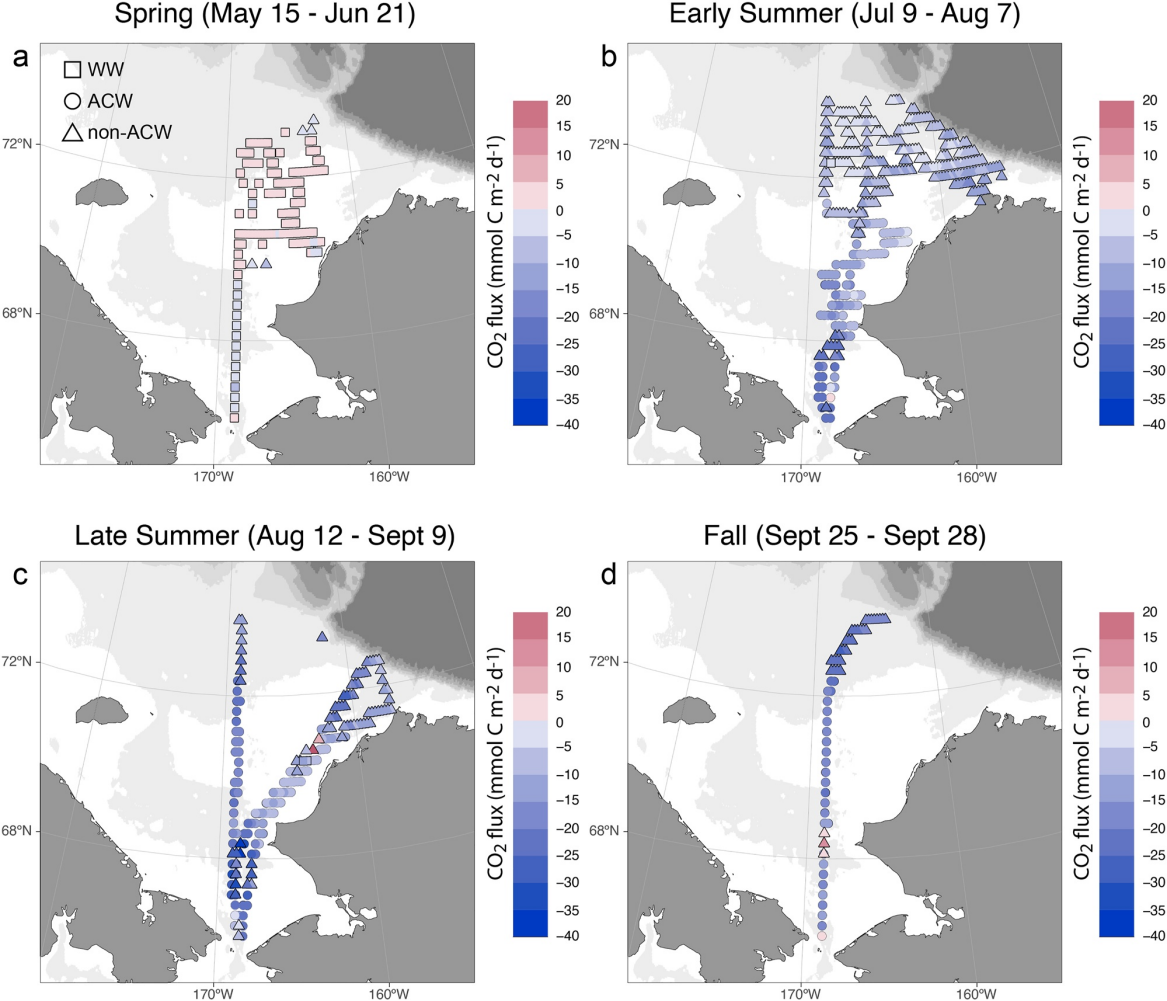
Seasonal variation in air‐sea CO_2_ flux in the Chukchi Sea in 2014. CO_2_ flux were derived from gridded *p*CO_2_ data (0.25° × 0.25°). Negative values of CO_2_ flux values indicate that CO_2_ uptake from the atmosphere. Square, circle and triangle represent winter water (Newly‐Ventilated Winter Water and remnant winter water), Alaska coastal water (ACW and river water), and non‐Alaska coastal water (Bering summer water and meltwater), respectively.

Seasonal evolution of water masses affects primary production and subsequently air‐sea CO_2_ exchange. To investigate the correlation between water mass change and ocean CO_2_ uptake, we first illustrate the relationship between water mass properties and seasonal change in sea surface *p*CO_2_ in T/S diagrams (Figure 10). In spring, most of the net change in *p*CO_2_ was negative since a high *p*CO_2_ value (i.e., 538 μatm) in the earlier winter water under the ice was used as the reference value (Figure 10a). The high *p*CO_2_ values that exceeded atmospheric *p*CO_2_ were concentrated within the cold and salty NVWW and RWW beneath the ice, which was resulted from remineralization in and vertical mixing of waters from the previous winter (Figure 10a). As sea ice started to retreat in late spring and early summer, the dilution by meltwater and strong biological CO_2_ uptake led to extremely low (<100 μatm) *p*CO_2_ along the ice edge (Figures 3c and 3f) with the largest seasonal reduction in the early‐season meltwater (Early‐MW; Figure 10b). These two processes primarily triggered the transition from a CO_2_ source to a sink within a few weeks. During this period, the average CO_2_ flux changed from 1.1 ± 1.1 mmol m^−2^ d^−1^ (from sea to air, or a source) to −7.2 ± 3.4 mmol m^−2^ d^−1^ (from air to sea, or a sink) in the northern Chukchi Sea (Figures 11a and 11b; Table 4). As the season progressed, the surface NVWW and RWW were largely transformed or replaced by summer Pacific waters (i.e., ACW and BSW). Consequently, the nutrient‐rich BSW sustained strong primary production and resulted in another extremely strong CO_2_ uptake center in the southern Chukchi Sea north of the Bering Strait (∼68°N, Figure 11c), whereas the warmer (SST > 8°C), relatively nutrient‐poor ACW had relatively weaker CO_2_ uptake along the eastern coastal area (Figure 11c). We noticed a small area of CO_2_ efflux in the eastern Chukchi Sea in late summer (Figure 11c) and an area of CO_2_ source water in the BSW regime in the southern Chukchi Sea in fall (∼69°N; Figure 11d). These high *p*CO_2_ surface waters were likely due to local vertical mixing as energetic currents in this area of shallow depths (<50 m) could break down stratified vertical structure and bring high *p*CO_2_ bottom water up to the surface (Brown et al., 2015). The regional mean CO_2_ flux in late summer and fall were estimated to range from −18.3 ± 8.9 to −10.5 ± 8.4 mmol m^−2^ d^−1^ in the southern Chukchi Sea and from −13.3 ± 6.6 to −21.1 ± 2.9 in the northern Chukchi Sea (Table 4).

Decomposition of the seasonal change in *p*CO_2_ provides more insight into the controlling drivers. Between spring and early summer, the largest reduction in *p*CO_2_ was observed in the early‐season MW and BSW, while the *p*CO_2_ reduction in the ACW was relatively less (Figure 10b). Specifically, the warm ACW and river runoff have the largest thermal effect on changing *p*CO_2_; a warming of more than 10°C in SST along could potentially increase *p*CO_2_ by ∼300 μatm (Figure 10f). In contrast, thermal effects were the relatively weaker driver for *p*CO_2_ change in both the BSW and MW (Figure 10f). In addition, the nutrient‐rich BSW provided a continued nutrient supply for sustaining primary production and biological CO_2_ drawdown along the Central Channel pathway. The strong reduction of *p*CO_2_ within BSW and MW domains (Figure 10j) even exceeded the increase induced by warming (Figure 10f), resulting in a net decrease in *p*CO_2_ and a strong CO_2_ sink (Figures 10b and 11b). Similar patterns existed in the following seasons (Figures 10, 11c and 11d)

As the CO_2_ uptake mainly occurred within Pacific Summer Water and thermal and non‐thermal effects had different impacts on CO_2_ flux in different water masses, we estimated CO_2_ fluxes separately in two types of water masses: the ACW (including RW) and non‐ACW (including BSW and MW but excluding NVWW and RWW). We found that the CO_2_ uptake potential in the nutrient‐rich non‐ACW was significantly larger than that in the relatively nutrient‐poor ACW in the southern Chukchi Sea during summer months (Table 4). Such difference became less contrasting because these two water masses tended to have more similarities as they were modified by mixing, meltwater input, and other local processes in the northern Chukchi Sea (Table 4). This finding has important implications for CO_2_ uptake in the Chukchi Sea. Woodgate (2018) found that annual inflow of Pacific waters has increased by more than 50% from 1990 to 2015, but there was no significant trend in annual mean flow in the ACC. Corlett and Pickart (2017) also confirmed that BSW (non‐ACW) increased relative to ACW on the Chukchi slope from 2002 to 2004 to 2009–2014. Thus, non‐ACW was likely responsible for the increase in total annual flow. If this is confirmed to be the case, we suggest that the Chukchi Sea will be a greater CO_2_ sink in the future, as non‐ACW will play a bigger role in CO_2_ uptake.

### 4.2 Water Mass Evolution and Net Community Production

**Figure 12:**
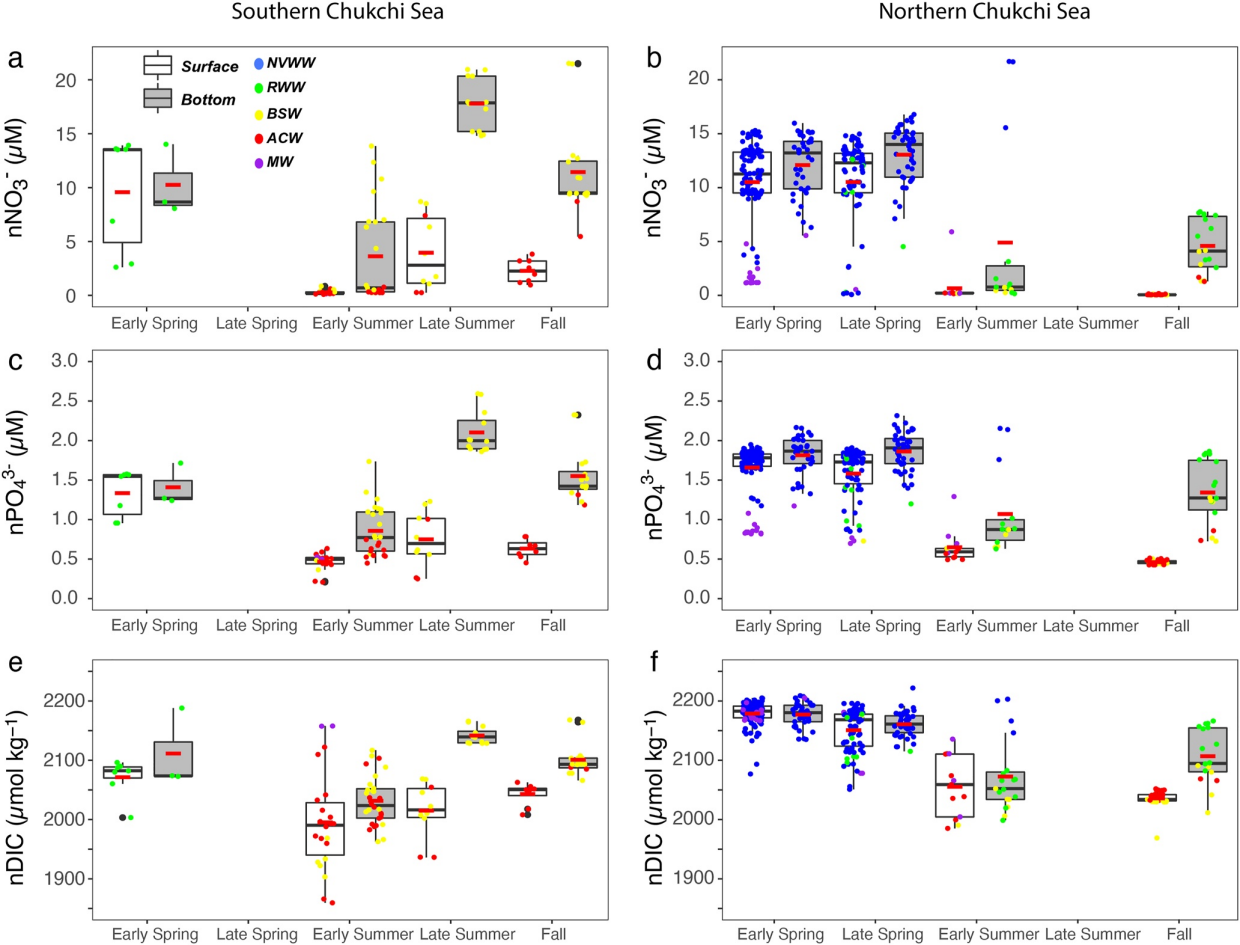
Seasonal variations of ${\text{nNO}}_{3}^{-}$, ${\text{nPO}}_{4}^{3-}$, and nDIC and in the southern (<69.5°N; a, c, and e) and northern Chukchi Sea (>69.5°N; b, d and f). White and gray boxplots indicate data collected in the surface and bottom layers, respectively. Black and red bars within boxplots indicate medians and means, respectively. Individual measurements are also shown and color‐coded by different water masses.

Spatial and temporal variations in water mass distributions substantially affect primary production in the Chukchi Sea during the growing season. In spring, both the southern and northern Chukchi Sea were largely occupied by the winter water (NVWW and RWW), which provided sufficient nutrients for phytoplankton to bloom (Figures 12a–12d; Arrigo et al., 2017). From late spring to early summer, winter waters were gradually transformed to or replaced by the warmer summer waters (BSW and ACW), particularly in the surface layer (Figure 12). At the same time, both normalized nitrate (n${\text{NO}}_{3}^{-}$), phosphate (n${\text{PO}}_{4}^{3-}$), and DIC (nDIC) decreased rapidly as a result of strong consumption due to phytoplankton growth. In late summer and fall, only a small portion of RWW existed in the bottom layer in the northern Chukchi Sea, while BSW and ACW accounted for the majority of surface water over the entire Chukchi Sea (Figure 12). For the bottom layer, ${\text{NO}}_{3}^{-}$ and ${\text{PO}}_{4}^{3-}$ were replenished by a combination of advection of nutrient‐rich BSW and possible vertical mixing from the bottom layer. This input of ${\text{NO}}_{3}^{-}$ and ${\text{PO}}_{4}^{3-}$ exceeded the biological drawdown in the southern Chukchi Sea, which resulted in a net increase in ${\text{nNO}}_{3}^{-}$ and n${\text{PO}}_{4}^{3-}$ concentrations in both the surface and bottom layers (Figures 12a and 12c). However, in the northern Chukchi Sea, ${\text{nNO}}_{3}^{-}$ concentration remained depleted and n${\text{PO}}_{4}^{3-}$ was the same in the surface layer but both increased in the bottom waters (Figures 12b and 12d). The high residual ${\text{nNO}}_{3}^{-}$ and n${\text{PO}}_{4}^{3-}$ in the bottom water were likely the remaining within winter waters rather than BSW delivery (Zheng et al., 2021).

As primary production is the main driver modifying water column biogeochemical properties during the growing season, we estimated NCP using the observed drawdown of nutrients (n${\text{NO}}_{3}^{-}$ and n${\text{PO}}_{4}^{3-}$) and nDIC in the surface mixed layer. The spring concentrations of n${\text{NO}}_{3}^{-}$, n${\text{PO}}_{4}^{3-}$ and nDIC were used as an initial condition to determine the subsequent biological drawdown by NCP. As the largest changes were observed in the period between spring and early summer, which were also associated with the most evident water mass evolution, therefore, we will mainly focus on this period in the following discussion.

Although there were no observations in late spring in the southern Chukchi Sea (Figures 12a, 12c and 12e), a water mass analysis showing that the winter water still made up most of the southern Chukchi Sea during this period (Pacini et al., 2019). Based on that, we assume that water parcel in the southern Chukchi Sea between early spring and early summer was not greatly changed and it is still appropriate to estimate NCP based on the deficits of nutrients and nDIC. The decreases of nDIC in the surface mixed layer between spring and early summer were 79 ± 87 and 95 ± 51 μmol kg^−1^, respectively, in the southern and northern Chukchi Sea (Figures 12e and 12f; Table 5). After correcting for air‐sea gas exchange and CaCO_3_ formation and dissolution, these nDIC deficits yield mean NCP estimates of 38.4 ± 26.2 and 61.4 ± 20.0 mmol C m^−2^ d^−1^, respectively, in the southern and northern Chukchi Sea (Table 5). Our nDIC‐based NCP estimates in 2014 were comparable with NCP estimates made using a similar approach in early summer in 2004 in the northern Chukchi Sea (Mathis et al., 2009). During the same period, ${\text{nNO}}_{3}^{-}$ in the surface mixed layer was fully depleted over the entire Chukchi Sea (Figures 12a and 12b). Such ${\text{NO}}_{3}^{-}$ depletion was also observed in early summer in 2010 and 2011 (Brown et al., 2015; Lowry et al., 2015). The mean n${\text{NO}}_{3}^{-}$ deficits in 2014 were 10.24 ± 0.20 and 9.92 ± 1.57 μM in the southern and northern Chukchi Sea, respectively (Table 5), giving an NCP of 3.51 ± 0.07 and 5.06 ± 0.80 mmol N m^−2^ d^−1^ in the southern and northern Chukchi Sea, respectively (Table 5). Because the mean n${\text{PO}}_{4}^{3-}$ deficits were 1.19 ± 0.11 and 0.94 ± 0.21 μM in the southern and northern Chukchi Sea, respectively (Table 5), n${\text{PO}}_{4}^{3-}$‐based NCP was estimated to be 0.41 ± 0.04 and 0.48 ± 0.11 mmol P m^−2^ d^−1^ in the southern and northern Chukchi Sea, respectively (Table 5). Given a M C:N:P uptake ratio of 106:16:1 (Redfield, 1958), n${\text{NO}}_{3}^{-}$‐based NCP was estimated to be 23.2 ± 0.5 and 33.4 ± 5.3 mmol C m^−2^ d^−1^ in the southern and northern Chukchi Sea. The analogous rates for n${\text{PO}}_{4}^{3-}$‐based NCP were 43.3 ± 4.0 and 50.9 ± 11.3 mmol C m^−2^ d^−1^, respectively. Thus, the nDIC‐based NCP was significantly higher than the NCP derived from n${\text{NO}}_{3}^{-}$ consumption by 66%–84% and was close to or slightly higher than n${\text{PO}}_{4}^{3-}$‐based NCP (Table 5). Although river runoff or precipitation may dominate endmembers in some locations and give different NCP estimates, it still cannot reconcile discrepancy in NCPs (Text S2 and Table S2 in Supporting Information S1). Therefore, other processes must add nutrients or remove DIC, or a novel, unaccounted for mechanism is required to explain this disparity.

N_2_ fixation could be one possible process providing new nitrogen to the system and would reconcile the inconsistency between the NCP estimates (Park et al., 2008). Recent studies have shown that N_2_ fixation can occur at low temperatures and high latitudes (Harding et al., 2018; Mills et al., 2018; Shiozaki et al., 2018; Sipler et al., 2017), which challenges the historical views that N_2_ fixation is a warm‐water process constrained to subtropical and tropical oligotrophic areas (von Friesen and Riemann, 2020; Zehr & Capone, 2020). Shiozaki et al. (2018) reported the N_2_ fixation rates of 0.08–3.60 nmol N L^−1^ d^−1^ during late summer in 2015 in the Chukchi Sea, which is equivalent to 0.002–0.09 mmol N m^−2^ d^−1^ integrated over the top 25 m. Accounting for such a new source of nitrogen (using the upper limit), n${\text{NO}}_{3}^{-}$‐based NCP would slightly increase by only ∼2%, to 3.60 ± 0.07 and 5.15 ± 0.80 mmol N m^−2^ d^−1^ in the southern and northern Chukchi Sea, respectively (Table 5). Thus, N_2_ fixation cannot explain the inconsistencies in NCP estimates based on the deficits of nDIC and n${\text{NO}}_{3}^{-}$.

CaCO_3_ formation or bio‐calcification would also contribute to DIC removal in the water column. However, a net increase in nTA in both southern and northern Chukchi Sea (Table 5) suggests that CaCO_3_ dissolution (e.g., mineral ikaite in ice) may dominate TA dynamics, which actually adds more DIC in the water column. By comparing the NCP results of with or without CaCO_3_ dissolution correction, we further estimated that CaCO_3_ dissolution accounted for ∼2.6% and 11.0% NCP‐_nDIC based_ in the southern and northern Chukchi Seas, respectively, during the period between spring and early summer. Thus, CaCO_3_ dissolution cannot explain the inconsistencies in NCP estimates either.

An alternative explanation is that, during intensive primary production, phytoplankton carbon and nutrient uptake in the Chukchi Sea are non‐Redfieldian; DIC uptake exceeds that expected for the observed N and P uptake. Based on deficits of nDIC and n ${\text{PO}}_{4}^{3-}$, we found that C:P uptake ratio only slightly deviated from the Redfield ratio around 94.0–128.0 (Table 5). Since the remaining ${\text{PO}}_{4}^{3-}$ (∼0.5 μM) would not limit phytoplankton growth, the inconsistency in NCP must be attributable to a non‐Redfield C:N uptake behavior. We found that phytoplankton growth in a N‐limited surface water led to a C:N uptake ratio of about 10.9–12.1 (Table 5), which is close to the measurements of the particulate organic C:N ratio in the Chukchi Sea in the summer of 2002 (C:N ratio >9; Bates et al., 2005), but much higher than the Redfield ratio and the observed annual mean of particulate organic C:N ratio (∼6.4; Frigstad et al., 2014). We attribute this disagreement to a rapid seasonal change in the phytoplankton C:N uptake ratio or an ecological response. The high NCP during late spring throughout early summer makes the Chukchi Sea an extremely ${\text{NO}}_{3}^{-}$‐limited ecosystem (Brown et al., 2015; Codispoti et al., 2013; Mills et al., 2015; Zheng et al., 2021), resulting in a higher C:N uptake ratio and hence a higher particulate organic C:N ratio (Bates et al., 2005). In contrast, the C:N uptake ratio is relatively low (close to the Redfield ratio) in early spring (Bates et al., 2005) and fall (Frigstad et al., 2014) when ${\text{NO}}_{3}^{-}$ is replenished.

Additional evidence for C:N variability comes from water column‐integrated NCP estimates. As phytoplankton growth and nutrient consumption may erode the nitricline, NCP calculated from surface water depletion of ${\text{NO}}_{3}^{-}$ and DIC probably underestimates the true NCP or overestimates the C:N uptake ratio, we computed depth‐integrated NCP for the entire water column. Previously, phytoplankton growth was observed in bottom waters in the southern Chukchi Sea in summers of 2010 and 2011 (Brown et al., 2015). In 2014, we observed excessive consumption of ${\text{NO}}_{3}^{-}$ in bottom waters not only in the southern Chukchi Sea, but also in the northern part (Figures 7h and 12b). The mean depth‐integrated NCP in the southern and northern Chukchi Sea derived from nDIC changes were 65.4 ± 46.7 and 98.7 ± 55.8 mmol C m^−2^ d^−1^, and NCP derived from n ${\text{NO}}_{3}^{-}$ changes were 5.3 ± 1.5 and 8.4 ± 4.9 mmol N m^−2^ d^−1^, respectively. These proportional increases in NCP estimates yield a C:N of 11.8–12.2, similar to that from the mixed layer analysis (Table S1 in Supporting Information S1), which confirms that C:N uptake ratio in the period between late spring and early summer was much higher than the Redfield ratio (C:N ∼ 6.6). Given that ${\text{nNO}}_{3}^{-}$ was still depleted in late summer and fall in the northern Chukchi Sea (Figure 12b), we further suggest that the C:N uptake ratio would remain high in the northern Chukchi Sea, while the C:N uptake ratio may return to the canonical Redfield ratio in the southern Chukchi Sea in the later season due to ${\text{NO}}_{3}^{-}$ replenishment exceeding consumption (Frigstad et al., 2014; Zheng et al., 2021).

### 4.3 Flexible Stoichiometric C:N Uptake Ratio Enhances Air‐Sea CO_2_ Uptake

The Chukchi Sea has been identified as a N‐limited ecosystem during the growing season (Brown et al., 2015; Codispoti et al., 2013; Mills et al., 2015; Zheng et al., 2021; Zhuang et al., 2020). Thus, DIC assimilation and associated NCP calculation is directly linked to the uptake of the most limited nutrient (${\text{NO}}_{3}^{-}$) and an assumption of a fixed C:N uptake ratio (Arrigo et al., 2017; Hansell et al., 1993). However, these assumptions are not always valid, especially when phytoplankton are experiencing N‐limitation, because the internal C:N ratio of phytoplankton can deviate from the Redfield ratio (Finkel et al., 2010; Spilling et al., 2015) and the produced carbon‐rich DOM can contribute a significant fraction of NCP (Baetge et al., 2020; Bif & Hansell, 2019). For example, when nutrients are sufficient, phytoplankton can assimilate and store extra nutrients for future use (i.e., luxury consumption; Elrifi & Turpin, 1985; Sommer, 1991). When nutrients in the internal pool or ambient water are not adequate, phytoplankton may release the fixed carbon as extracellular dissolved organic carbon (DOC) (Myklestad, 1995) or produce transparent exopolymeric particles (Mari et al., 2001; Vernet et al., 1998) and carbon‐rich organic matter (Humphreys et al., 2019). From a biogeochemical perspective, this non‐Redfield C:N uptake by phytoplankton may greatly impact seasonal variation in sea surface *p*CO_2_ and air‐sea CO_2_ flux. For example, Fransner et al. (2018) found that CO_2_ uptake from the atmosphere can be underestimated by 50% in the northern Baltic Sea if a fixed Redfield ratio is used to determine carbon assimilation. However, there is little evidence for such a mechanism operating in the polar regions.

**Figure 13:**
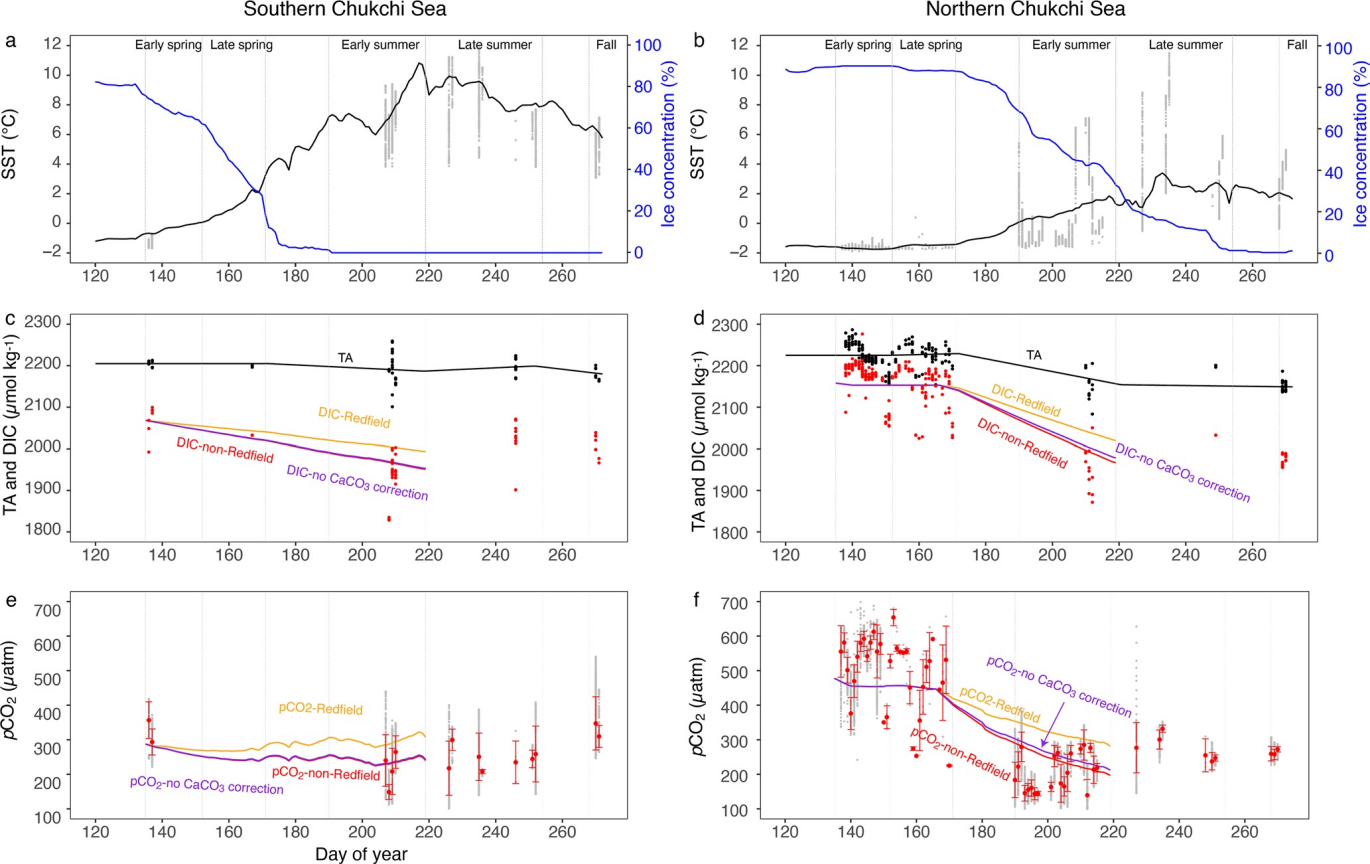
Observed and modeled seasonal variations in (a, b) Sea surface temperature (SST) and ice concentration (c, d) TA and dissolved inorganic carbon (DIC), and (e, f) *p*CO_2_ in the southern Chukchi Sea (left column) and the northern Chukchi Sea (right column). The yellow lines are model results with fixed C:N uptake ratio (Redfield ratio). The red and purple lines indicate the model results with non‐Redfield C:N uptake ratio with and without CaCO_3_ dissolution correction, respectively. Black and red dots in c and d are discrete samples of TA and DIC, respectively. Gray dots are underway measurements of SST (a, b) and *p*CO_2_ (e, f). Daily means with error bar (± standard deviation) of underway *p*CO_2_ are presented in e and f. Five periods, including early and late spring, early and late summer, and fall are indicated by vertical dashed lines.

To demonstrate how a flexible C:N uptake stoichiometry by phytoplankton and possible CaCO_3_ dissolution during active ice melting affect the air‐sea CO_2_ flux, we used a box model to reproduce sea surface *p*CO_2_ in the Chukchi Sea from spring to early summer in 2014. Three simulations were performed; scenario 1 with a non‐Redfield C:N uptake ratio corrected for CaCO_3_ dissolution, scenario 2 with a non‐Redfield C:N uptake ratio without correcting for CaCO_3_ dissolution and scenario 3 with a fixed Redfield C:N ratio (Figure 13). In the simulation with a non‐Redfield C:N uptake stoichiometry (scenario 1 & 2), more efficient DIC drawdown led to a lower DIC concentration during early summer when compared to the Redfield ratio scenario, which results in better agreement with observations in both the southern and northern Chukchi Sea (Figures 13c and 13d). Furthermore, the non‐Redfield stoichiometry approach better reproduces observations of *p*CO_2_ during this intensive growing season (Figures 13e and 13f), whereas carbon fixation in the fixed‐stoichiometry (Redfield ratio) simulation is not efficient enough to counteract the effects of warming and air‐sea CO_2_ exchange on sea surface *p*CO_2_. More importantly, the higher carbon fixation with a non‐Redfield C:N uptake ratio, compared to the fixed Redfield uptake ratio, enhances air‐sea CO_2_ uptake in the Chukchi Sea. The net CO_2_ uptake from the atmosphere in the non‐Redfield stoichiometry scenario over the simulation period (day 136 to day 219 for the southern Chukchi Sea and day 167 to day 219 for the northern Chukchi Sea) is estimated to be 751 mmol C m^−2^ and 343 mmol C m^−2^, respectively, in the southern and northern Chukchi Sea, which are 42% and 85% higher than CO_2_ uptake in the fixed‐stoichiometry scenario (528 mmol C m^−2^ and 185 mmol C m^−2^). Thus, we conclude that about 30%–46% of CO_2_ uptake during this intensive growing season is supported by phytoplankton stoichiometric flexibility. By comparing the results of scenarios 1 and 2, we further quantified that the correction of CaCO_3_ dissolution during ice melting from spring to early summer accounted for ∼1% and 9% CO_2_ uptake in the southern and northern Chukchi Sea, respectively, which is a significant fraction of NCP estimate. The difference in the net uptake of atmospheric CO_2_ between these simulations could increase as nutrient‐limited conditions and high C:N uptake mechanisms extend to a longer growing season (e.g., late summer and fall).

## 5 Summary

Seasonality of biogeochemical properties determines how an ocean ecosystem responds to external and internal forcings. By synthesizing a rare data set of underway measurements and discrete samples collected in five consecutive cruises in 2014, we presented a complete seasonal cycle (covering spring through fall) of sea surface *p*CO_2_ and biogeochemical properties in the Chukchi Sea.

We first explored the dominant drivers of seasonal *p*CO_2_ change as water masses evolve, revealing that thermal and non‐thermal effects have different impacts on sea surface *p*CO_2_ and air‐sea CO_2_ fluxes in different water masses. The non‐ACW with stronger biological CO_2_ removal and a weaker warming effect has a stronger atmospheric CO_2_ uptake potential in summer months than ACW does. We suggest that the Chukchi Sea will become a greater CO_2_ sink in the future as the proportion of nutrient‐rich non‐ACW increases.

We then estimated NCP for the most intensive growing period (spring to early summer) using observed DIC and nutrient data, and found that carbon‐based NCP was consistently higher than ${\text{NO}}_{3}^{-}$‐based NCP by 66%–84%. We attributed this inconsistency in NCP estimates to a non‐Redfield uptake of carbon and nutrients.

To investigate this hypothesis, we performed two model simulations to test how a flexible stoichiometry of C:N uptake ratio can affect seasonal biogeochemical dynamics and air‐sea CO_2_ exchange. Comparing modeled results and observations, we show that a variable phytoplankton C:N stoichiometry is needed in order to better simulate and understand the seasonal biogeochemical dynamics and air‐sea CO_2_ exchange. In particular, this stochiometric flexibility in phytoplankton enables more efficient DIC‐fixation, which contributes about 30%–46% of CO_2_ uptake from atmosphere in the Chukchi Sea. These model results also have important implications for biological pump estimates in the Chukchi ecosystem and parametrizing C and N cycles in regional biogeochemical models.

## Conflict of Interest

The authors declare no conflicts of interest relevant to this study.

## Supporting information

Supporting Information S1

## Data Availability

All the data are archived in publicly accessible databases. The SOCAT data can be downloaded from https://www.socat.info/index.php/data-access/. The data of discrete sample of carbonate chemistry, nutrients, dissolved oxygen and Chl *a* are also archived in publicly accessible databases (https://arcticdata.io/catalog/view/doi%3A10.18739%2FA21C1TG6R; https://data.mendeley.com/datasets/dfpxxwm24c/2; and http://www.godac.jamstec.go.jp/darwin/cruise/mirai/mr14-05/e). The model simulation data is also accessible via https://data.mendeley.com/datasets/xhj79xjhpc/1.
