# Supplementary material for: Seasonal Water Mass Evolution and Non‐Redfield Dynamics Enhance CO2 Uptake in the Chukchi Sea
Source: J Geophys Res Oceans. 2022 Aug 4;127(8):e2021JC018326. doi: 10.1029/2021JC018326 (PMC9787980; doi:10.1029/2021JC018326)
Supplement: Supplementary file 1 — Supporting Information S1 [file JGRC-127-e2021JC018326-s001.docx]

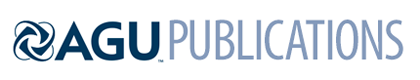


*Journal of Geophysical Research Oceans*

Supporting Information for

**Seasonal water mass evolution and non-Redfield dynamics enhance CO_2_ uptake in the Chukchi Sea**

Zhangxian Ouyang^1^, Andrew Collins^1,2^, Yun Li^1^, Di Qi^3,4^, Kevin R. Arrigo^5^, Yanpei Zhuang^3,6^, Shigeto Nishino^7^, Matthew P. Humphreys^8^, Naohiro Kosugi^9^, Akihiko Murata^10^, David L. Kirchman^1^, Liqi Chen^4^, Jianfang Chen^6^, Wei-Jun Cai^1^*

^1^ School of Marine Science and Policy, University of Delaware, Newark, Delaware, USA

^2^ NOAA Pacific Marine Environmental Laboratory, Seattle, WA, USA

^3^ Polar and Marine Research Institute, Jimei University, Xiamen, China

^4^ Key Laboratory of Global Change and Marine-Atmospheric Chemistry of Ministry of Natural Resources, Third Institute of Oceanography, MNR, Xiamen 361005, China

^5^ Department of Earth System Science, Stanford University, Stanford, CA 94305, USA

^6^ Key Laboratory of Marine Ecosystem Dynamics, Second Institute of Oceanography, Ministry of Natural Resources, Hangzhou 310012, China

^7^ Institute of Arctic Climate and Environment Research, Japan Agency for Marine-Earth Science and Technology (JAMSTEC), Yokosuka, Japan

^8^ NIOZ Royal Netherlands Institute for Sea Research, Department of Ocean Systems (OCS), Texel, the Netherlands

^9^ Meteorological Research Institute, Tsukuba, Japan

^10^ Global Ocean Observation Research Center, Research Institute for Global Change, Japan Agency for Marine-Earth Science and Technology (JAMSTEC), Yokosuka, Japan

*Corresponding author, email: [wcai@udel.edu](mailto:wcai@udel.edu)

**Contents of this file**

Text S1 to S2

Figures S1

Tables S1 to S2

**Text S1. End-member mixing analysis**

To identify the spatial variation in different water masses on the Chukchi Sea, we employ a three-endmember mixing model to estimate the fractions of each water mass (ice melt water, river water, and seawater) in each sample. This method has been employed by several other investigators for similar studies in the Arctic Ocean (e.g., Anderson et al., 2004; Yamamoto-Kawai et al., 2005 and 2008):

f_SIM_ + f_RW_ + f_SW_ =1 (1)

f_SIM_S_SIM_ + f_RW_S_RW_ + f_SW_S_SW_ =S_obs_ (2)

f_SIM_TA_SIM_ + f_RW_TA_RW_ + f_SW_TA_SW_ =TA_obs_ (3)

where f denotes fraction, SIM denotes sea-ice meltwater, RW denotes river water, and SW denotes Pacific Water. S_obs_ and TA_obs_ are the observed salinity and TA, respectively. The end-members of Pacific Water, S = 32.6 and TA = 2211 μmol kg^-1^, are selected from averaged values of upwelled Bering Sea slope water during summer 2014 as this is the main source of waters entering the Arctic Ocean through the Bering strait. The end-member values for ice meltwater (S = 5, TA = 460 μmol kg^-1^) are based on values reported by Rysgaard et al. (2007), while the end-member values for river water (S = 0; TA = 1600 μmol kg^-1^) are similar to those reported by Cooper et al. (2008) for the flow-weighted averages of the Yukon and Mackenzie rivers, which most likely had potential influence on our measurements due to their proximity to our study area.

As we focused on the period between spring and early summer, which have the largest change in biogeochemical properties, we presented here the meltwater and river water fractions along several sections on the Chukchi Shelf during early summer in 2014 (Fig. S1). Similar latitudinal pattern has been seen in the fractions of meltwater and river water (Fig. S1 a and b). However, most of river water was constrained in the eastern Chukchi Sea along the coastal areas (Fig. S1 f, g, and h), which indicates the river discharge impact in spring and early summer was limited to the estuarine and nearshore areas and that the salinity change over the shelf was mainly due to ice melt dilution.

**Text S2. Impact of selection of freshwater endmember on NCP calculation**

As the Chukchi Sea shelf water was influenced by freshwater (meltwater, river runoff and precipitation), the selection of freshwater endmember for calculating salinity-normalized DIC and TA may affect the NCP estimates. Therefore, we performed sensitivity tests to examine how NCP estimates changes based on three typical scenarios. For the first scenario, we assumed that the observed water is the mixture of Pacific water and meltwater as we presented in the main text. For the second and third scenarios, we assumed that the observed water is the mixture of Pacific water and river runoff, and precipitation, respectively. These three scenarios set the boundary for most of the water observed in the Chukchi Sea as it is the mixture of Pacific seawater, meltwater, river runoff and precipitation. Thus, for the second scenario, salinity-normalized DIC and TA are calculated similarly (Equ (8) and (9)) but using river runoff endmember, which is S = 0, TA = 1600 μmol kg^-1^ and DIC = 1500 μmol kg^-1^ (Cooper et al., 2008; Guo et al., 2012). The endmember for precipitation is set as S= 0, TA = 0 μmol kg^-1^ and DIC = 0 μmol kg^-1^. We showed the estimated NCP in Table S2. We found that although NCP_nDIC_ estimate based on the river water endmember is higher than the results of meltwater and precipitation endmembers, it cannot reconcile discrepancy in NCPs (Table S2), and it won’t change the mechanism of non-Redfield C:N uptake we proposed here.


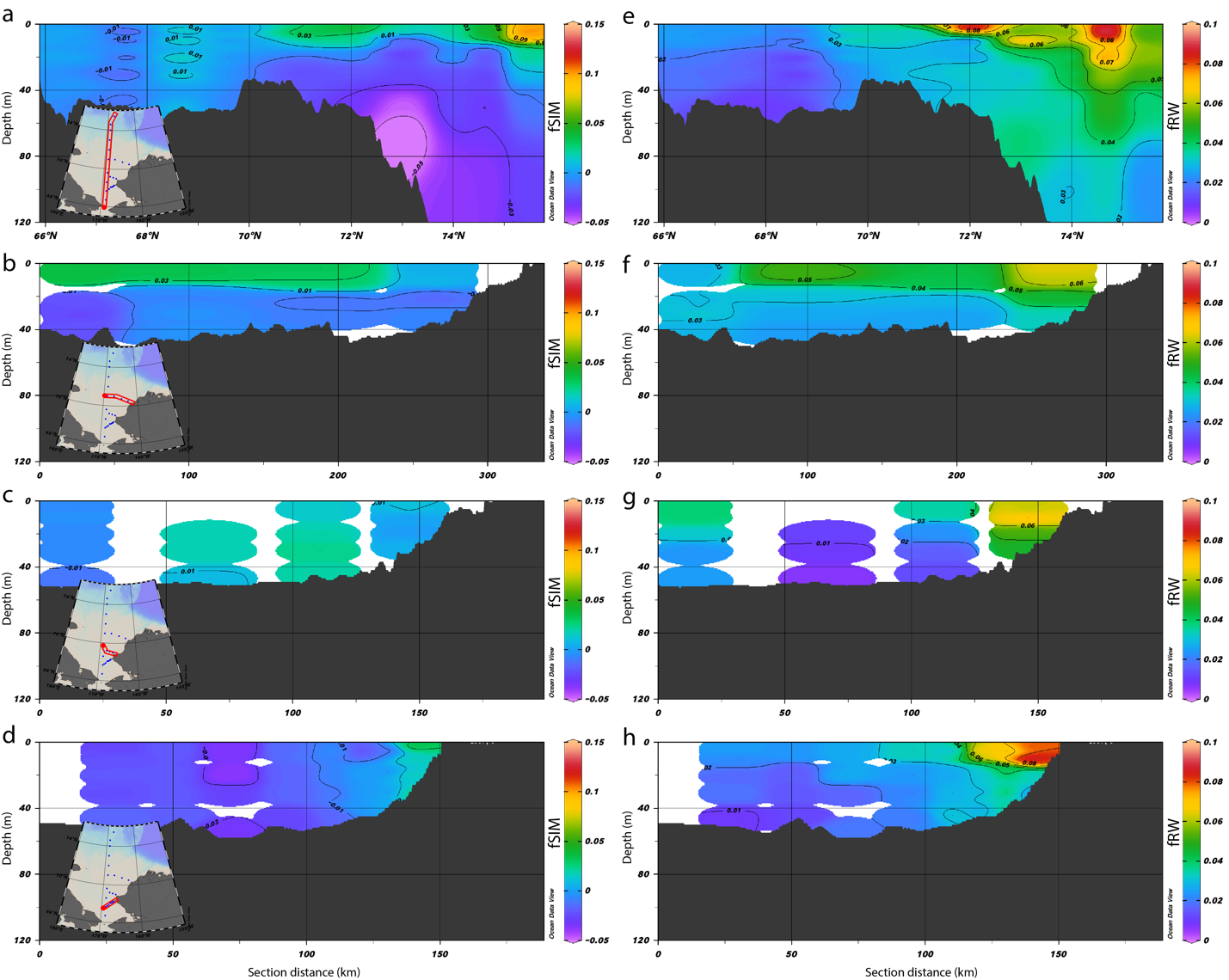


Figure S1. Vertical sections of fractions of sea ice meltwater (fSIM, a-d) and river water (fRW, e-h) on the Chukchi shelf during early summer 2014.

Table S1. NCP estimates based on the water column integrated change in nDIC and n$\mathrm{NO}_{3}^{-}$ for the period between the late spring and the late summer of 2014 in the Chukchi Sea. N-based NCP was converted to carbon (C) units assuming a Redfield molar C:N uptake ratio of 106:16.

| Location | Growing season | NCP (nDIC based) | NCP (n$\mathrm{NO}_{3}^{-}$ based) | | C:N uptake ratio |
| --- | --- | --- | --- | --- | --- |
|  | day | mmol C m^-2^ d^-1^ | mmol N m^-2^ d^-1^ | mmol C m^-2^ d^-1^ |  |
| Southern | 73 | 65.4$\pm$46.7 | 5.3$\pm$1.5 | 35.2$\pm$10.1 | 12.2 |
| Northern | 49 | 98.7$\pm$55.8 | 8.4$\pm$4.9 | 55.4$\pm$32.1 | 11.8 |

Table S2. NCP estimates based on the changes in nDIC and n$\mathrm{NO}_{3}^{-}$ in the surface mixed layer for the period between the spring and the early summer of 2014 in the Chukchi Sea. Three scenarios of mixing are tested: salinity-normalization of DIC and TA are based on: a, the mixing of Pacific water and meltwater; b, the mixing of Pacific water and river water; c, the mixing of Pacific water and precipitation. N-based NCP was converted to carbon (C) units assuming a Redfield molar C:N uptake ratio of 106:16. C:N uptake ratios in three scenarios are calculated as NCPnDIC : NCPn$\mathrm{NO}_{3}^{-}$.

| Location | Growing season | NCP (nDIC based)^a^ | NCP (nDIC_r_ based)^b^ | NCP (nDIC_S0_ based)^c^ | NCP (n$\mathrm{NO}_{3}^{-}$ based) | | C:N uptake ratio | | |
| --- | --- | --- | --- | --- | --- | --- | --- | --- | --- |
|  | day | mmol C m^-2^ d^-1^ | mmol C m^-2^ d^-1^ | mmol C m^-2^ d^-1^ | mmol N m^-2^ d^-1^ | mmol C m^-2^ d^-1^ | a | b | c |
| Southern | 73 | 38.4 $\pm$ 26.2 | 46.8 $\pm$ 19.7 | 39.6 $\pm$ 26.3 | 3.51$\pm$ 0.07 | 23.2 $\pm$ 0.5 | 10.9 | 13.3 | 11.3 |
| Northern | 49 | 61.4 $\pm$ 20.0 | 81.9 $\pm$ 20.2 | 61.2 $\pm$ 20.3 | 5.06 $\pm$ 0.80 | 33.4 $\pm$ 5.3 | 12.1 | 16.2 | 12.1 |

^a^ The scenario of mixing of Pacific water and meltwater.

^b^ The scenario of mixing of Pacific water and river water.

^c^ The scenario of mixing of Pacific water and precipitation.
